# Lumbrokinase Extracted from *Earthworms* Synergizes with Bevacizumab and Chemotherapeutics in Treating Non-Small Cell Lung Cancer by Targeted Inactivation of BPTF/VEGF and NF-κB/COX-2 Signaling

**DOI:** 10.3390/biom14070741

**Published:** 2024-06-23

**Authors:** Chunyu Hua, Ziyue Guo, Meng Dai, Jie Zhou, Hanxiao Ge, Guoqing Xue, Fahui Xu, Liyuan Ru, Kuan Lv, Guohui Zhang, Lina Zheng, Meiyi Wang, Yun Teng, Wendan Yu, Wei Guo

**Affiliations:** 1 Institute of Cancer Stem Cells, Dalian Medical University, Dalian 116044, China; huacy@dmu.edu.cn (C.H.); guo_ziyue18@163.com (Z.G.); zhouj08@dmu.edu.cn (J.Z.); gehx@dmu.edu.cn (H.G.); xuegq01@dmu.edu.cn (G.X.); ruly01@dmu.edu.cn (L.R.); lvk@dmu.edu.cn (K.L.); zhanggh01@dmu.edu.cn (G.Z.); zhengln@dmu.edu.cn (L.Z.); wangmy06@dmu.edu.cn (M.W.); 2 Dalian Municipal Central Hospital, Dalian University of Technology, Dalian 116044, China; 18340810857@163.com; 3 The Second Clinical College, Dalian Medical University, Dalian 116044, China; xufahui123@outlook.com; 4 The Second Affiliated Hospital, Dalian Medical University, Dalian 116044, China; tengy@dmu.edu.cn

**Keywords:** lumbrokinase, BPTF, VEGF, NF-κB, COX-2, angiogenesis, chemoresistance

## Abstract

As a kind of proteolytic enzyme extracted from *earthworms*, lumbrokinase has been used as an antithrombotic drug clinically. Nevertheless, its potential in anti-cancer, especially in anti-non-small cell lung cancer (NSCLC), as a single form of treatment or in combination with other therapies, is still poorly understood. In this study, we explored the anti-tumor role and the responsive molecular mechanisms of lumbrokinase in suppressing tumor angiogenesis and chemoresistance development in NSCLC and its clinical potential in combination with bevacizumab and chemotherapeutics. Lumbrokinase was found to inhibit cell proliferation in a concentration-dependent manner and caused metastasis suppression and apoptosis induction to varying degrees in NSCLC cells. Lumbrokinase enhanced the anti-angiogenesis efficiency of bevacizumab by down-regulating BPTF expression, decreasing its anchoring at the *VEGF* promoter region and subsequent VEGF expression and secretion. Furthermore, lumbrokinase treatment reduced IC50 values of chemotherapeutics and improved their cytotoxicity in parental and chemo-resistant NSCLC cells via inactivating the NF-κB pathway, inhibiting the expression of COX-2 and subsequent secretion of PGE2. LPS-induced NF-κB activation reversed its inhibition on NSCLC cell proliferation and its synergy with chemotherapeutic cytotoxicity, while COX-2 inhibitor celecoxib treatment boosted such effects. Lumbrokinase combined with bevacizumab, paclitaxel, or vincristine inhibited the xenograft growth of NSCLC cells in mice more significantly than a single treatment. In conclusion, lumbrokinase inhibited NSCLC survival and sensitized NSCLC cells to bevacizumab or chemotherapeutics treatment by targeted down-regulation of BPTF/VEGF signaling and inactivation of NF-κB/COX-2 signaling, respectively. The combinational applications of lumbrokinase with bevacizumab or chemotherapeutics are expected to be developed as promising candidate therapeutic strategies to improve the efficacy of the original monotherapy in anti-NSCLC.

## 1. Introduction

According to statistics from the American Cancer Society, an estimated 238,340 people will be diagnosed with lung cancer, and 127,070 people will die from the disease in the United States in 2023, with approximately 350 people dying each day from lung cancer, which is almost 2.5 times more than the number of people who die from colorectal cancer, the second leading cause of cancer death overall [1]. Thus, lung cancer has become the leading cause of cancer-related death worldwide, both in the male and female populations. The overall mortality trends of cancer are largely driven by lung cancer [2]. Due to the difficulty of detection and diagnosis at the early stage and the frequent occurrence of metastasis and invasion of the disease, most lung cancer patients are generally diagnosed in an advanced stage [3]. Depending on histopathology, about 80% of lung cancers are non-small cell lung cancers (NSCLCs) [4]. At present, the treatments for NSCLC mainly include surgical resection, chemotherapy, radiotherapy, targeted therapy, and immunotherapy [5]. The clinical implementation of molecular targeted therapy and immunotherapy for tumors has greatly changed the therapeutic landscape of advanced NSCLC and has improved the outcomes of patients markedly in the past two decades [6]. However, the inevitable toxic side effects and the development of therapeutic resistance greatly limit the efficacy of these treatments and eventually lead to disease progression [7]. This is why lung cancer patients, especially advanced NSCLC patients, show a comparatively high mortality. Hence, it is highlighted and of great significance to deeply understand and decipher the mechanism of the occurrence and progression of NSCLC, in particular, the molecular mechanisms of therapy resistance production and development, and based on this, find new adjuvant therapeutic drugs and provide rational combination therapy strategies.

Tumor progression is often accompanied by the ingrowth of blood vessels, which is a need for malignant cells to have access to the circulation system to obtain oxygen and nutrients and to thrive [8]. Therefore, tumor angiogenesis plays a key role in tumor growth and metastasis [9,10]. In recent years, targeted inhibition of angiogenic signaling pathways or angiogenic genes has become research hotspots for tumor prevention and treatment [11]. The current anti-angiogenic drugs are mainly divided into two categories, namely monoclonal antibodies targeting VEGF or VEGFR and small molecule tyrosinase inhibitors targeting VEGFR [12]. The VEGF family includes VEGF-A, VEGF-B, VEGF-C, VEGF-D, VEGF-E, PIGF-1, and PIGF-2, of which VEGF-A is by far the most effective vascular growth factor [13,14]. Anti-VEGF monotherapy, such as Bevacizumab, an approved anti-tumor angiogenesis drug targeting VEGF, has shown a definite anti-tumor effect in a variety of malignant tumors, including NSCLCs [15,16,17,18]. Nevertheless, an increased tumor aggressiveness after anti-angiogenic therapy and the resistance that comes with it have appeared inevitably [19]. Moreover, serious side effects are also caused by this therapy, including the significant bleeding clinically, and the formation of thrombus promoted by endothelial cell changes, cytokine release, and other cellular interactions, bringing thrombosis risk to patients [20,21]. Therefore, for this form of treatment, how to reduce its toxic side effects, delay the generation of drug resistance, and improve the efficacy of clinical use has become the top priority of current research.

Chemotherapy is still the main and cornerstone treatment form for lung cancer, including NSCLC [22]. As a kind of adjuvant therapy in patients with resected NSCLC, chemotherapy is delivered as palliative therapy in patients with advanced NSCLC, or as part of multimodality therapy in patients with locally advanced NSCLC. Nevertheless, cancer patients are very likely to develop drug resistance after chemotherapy for a certain period of time and have to discontinue this treatment, causing the relapse and metastasis of the disease finally, although it can relieve cancer-related symptoms and increase the overall survival of patients [23]. Not only that, but chemical drugs are not specific to cancer cells, and they can also kill normal cells. Therefore, excessive chemotherapy might even shorten the survival time of cancer patients, including NSCLC patients. The combination application of chemotherapy with other treatment forms, such as immune checkpoint inhibitors and/or tyrosine kinase inhibitors, has been studied and the benefits have appeared clinically [24]. In line with this, other novel treatments, for example, traditional Chinese medicine anti-tumor therapy, might also be integrated into the overall treatment strategy that includes chemotherapy to improve the outcome of NSCLC patients.

Earthworm fibrinolytic enzyme (EFE), namely lumbrokinase, is a complex enzyme extracted from *earthworms* according to the established standardized extraction processes [25,26]. In addition to being a strong plasmin, lumbrokinase can also indirectly achieve anticoagulation by inhibiting platelet function [27,28,29]. For most cancer patients, especially advanced cancer patients, being hypercoagulable and prone to venous thromboembolism is a highly likely event [30]. There is accumulating evidence in vitro, clinically indicating that hypercoagulability is involved in tumor growth and metastasis stimulation, and the anticoagulants possess the potential to limit cancer survival and development [31,32]. Therefore, the use of lumbrokinase to prevent or treat cancer-associated thromboembolism is very reasonable. Its further use to inhibit tumor survival might also be taken into consideration, which means whether lumbrokinase treatment is beneficial for the outcome of tumor patients, especially NSCLC patients, deserves exploration. Several studies have indicated that lumbrokinase could inhibit the growth and metastasis of bladder cancer, gastric cancer, and hepatoma cells in vitro and in animal models [33], although it is still in the early stages of research. Furthermore, considering that the anticoagulant effect of lumbrokinase might possibly compensate for the risk of thrombosis caused by bevacizumab treatment in NSCLC patients, and its natural source, low toxicity, and certain anti-tumor activity might also possibly neutralize the toxic and side effects of chemotherapeutics and delay or weaken the emergence and development of chemoresistance, the potential of the combined treatment strategy including lumbrokinase and bevacizumab or lumbrokinase and chemotherapy might be highlighted for NSCLC patients. To systematically discover and confirm the option of such combinational treatment in NSCLC, in this study, we first perform a serial of functional experiments in vitro and confirm the anti-survival effect of lumbrokinase in NSCLC cells. We further find that the use of lumbrokinase down-regulates BPTF expression, alleviates the binding of *BPTF* at the specific promoter region of the *VEGF* gene, results in the decrease in VEGF expression, and finally synergizes with bevacizumab in NSCLC treatment. We also find that lumbrokinase treatment inactivates NF-κB signaling and alleviates the binding of p50/p65 at the *COX-2* promoter, leading to the down-regulation of COX-2 expression, and eventually sensitized NSCLC cells to chemotherapy. To sum up, our study aims to explore the anti-tumor role of lumbrokinase and the responsive molecular mechanisms in suppressing NSCLC angiogenesis and chemoresistance development, and based on this, to propose the possibility of lumbrokinase combined with bevacizumab or chemotherapeutics in treating NSCLC.

## 2. Materials and Methods

### 2.1. Cell Lines

Human non-small cell lung cancer cell lines H460, H1299, and A549, human embryonic lung fibroblasts HFL1, and human umbilical vein endothelial cell HUVEC were purchased from ATCC in the United States. Vincristine-tolerant and paclitaxel-tolerant H460 cells were constructed by our laboratory. H460, H1299, and HFL1 cells were cultured using RPMI-1640 medium supplemented with 10% FBS, and A549 cells were cultured using Dulbecco’s modified Eagle’s medium (DMEM) supplemented with 10% FBS. All cell lines were cultured in an incubator at 37 °C with 5% CO_2_.

### 2.2. Reagents and Antibodies

Vincristine (S9555), paclitaxel (S1150), and LPS (S7850) were purchased from Selleck Chemicals, Houston, TX, USA. Lumbrokinase (S10081) was purchased from Shanghai yuanye Bio-Technology Co., Ltd., Shanghai, China. Bevacizumab was purchased from Roche Pharmaceuticals, Basel, Switzerland. The antibody against BPTF for Western blot (ab72036) was purchased from Abcam (Cambridge, MA, USA) and for IHC (28016-1-AP) was purchased from Cell Signaling Technology (Danvers, MA, USA). The antibody against COX2 for Western blot (ab169782) was purchased from Abcam (Cambridge, MA, USA) and for IHC (sc-514489) was purchased from Santa Cruz Biotechnology (Dallas, CA, USA). The antibodies against p-c-Raf (Ser338) (#9427), p-MEK1/2 (Ser217/221) (#9154), p-p44/42 MAPK (Erk1/2) (Thr202/Tyr204) (#4370), p44/42 MAPK (Erk1/2) (#4695), p-p90rsk (Ser380) (#11989), p-p38 MAPK (Thr180/Tyr182) (#4511), p38 (#9212), PI3 Kinase p110γ (#5405), Phospho-PI3 Kinase p85 (Tyr458)/p55 (Tyr199) (#4228), PI3Kp85 (#4257), p-PDK1(Ser241) (#3438), p-Akt (Thr308) (#13038), p-GSK-3β (Ser9) (#9323), Akt (#4691), Caspase-9 (#9502), Ace-p53 (#2525), Vimentin (#5741), Snail (#3879), NF-κB Pathway Sampler Kit (#9936), and Histone H3 (#4499) were purchased from Cell Signaling Technology (Danvers, MA, USA). GAPDH (60004-1-Ig), PARP1 (13371-A-AP), Bcl-2 (26593-1-AP), Bax (50599-2-Ig), Actin (23660-1-AP), VEGF (19003-1-AP), and BPTF (28016-1-AP) were purchased from Proteintech Group (Wuhan, China). β-catenin (sc-7963), Ki67 (sc-23900), CD31 (sc-376764), p50 (sc-53744), p-p65 (sc-166748), and p65 (sc-8008) were purchased from Santa Cruz Biotechnology (Dallas, CA, USA).

### 2.3. Cell Viability Assay

Cells were seeded in 96-well plates with 6000 cells per well and cultured overnight. Then, the cells were treated with different concentrations of agents. After 48 h, MTT dilution was added and incubated for 3–4 h in the dark, and then DMSO was added after discarding the supernatant. Finally, the absorbance value at 490 nm was measured immediately after thorough dissolution.

### 2.4. Colony Formation Assay

Cells were seeded in six-well plates with 10,000 cells per well. When the cells grew to adhere, different concentrations of agents were added accordingly. After seven to ten days, the formed colonies containing more than 50 cells were stained with crystal violet and photographed.

### 2.5. Western Blot

The cells were collected after treatments with different concentrations of lumbrokinase for 48 h, and lysed by adding different volumes of protein lysate (including 1% protease inhibitor Cocktail) according to the amount of cell precipitates. After full lysis, the samples were centrifuged and the supernatant was collected into a new tube. The protein concentration of the lysate was calculated via BCA quantification assay (23228 Thermo Fisher, Waltham, MA, USA). According to the quantitative results, the same number of proteins from different samples were loaded into the SDS-PAGE gel and separated until the bromocresol blue ran to the bottom of the gel. Then, the proteins on the gel were transferred to the PVDF membrane that had been activated by methanol in advance. After the transfer, the PVDF membrane was immersed in 8% skimmed milk for 3–6 h. At the end of the closure, cold TBST was used to wash off excess skim milk on the surface of the PVDF membrane. The PVDF membrane was then incubated with the primary antibody overnight and then the excess primary antibody was washed off with cold TBST. After that, the PVDF membrane was incubated in a secondary antibody dilution and slowly shaken for 2 h at room temperature, and the excess secondary antibody was washed off with cold TBST. Finally, the proteins were detected by enhanced chemiluminescence.

### 2.6. RT-PCR

Firstly, total RNAs from the cell pellet were extracted using Trizol (Invitrogen Life Technologies, Carlsbad, CA, USA) and were reversed into cDNA using TransScript One-Step gDNA Removal and cDNA Synthesis SuperMix kit (#AT311, TransGen Biotech, Beijing, China) according to the instructions. Then, the target genes were determined by performing PCR using the obtained cDNA as a template, and the amplified products were run via agarose gel electrophoresis. The sequences of the primers for the target genes are as follows: *BPTF* (forward 5′-AATCGGAGAAGTCCAACGGG-3′; reverse 5′-TTGCCCTATGTGATGCCCAG-3′); *VEGF* (forward 5′-GTGTGCCCCTGATGCGATGCG-3′; reverse 5′-ACCGCCTCGGCTTGTCAC-3′); *COX2*(forward 5′-TACGACTTGCAGTGAGCGTC-3′; reverse 5′-AAGGGAGTCGGGCAATCATC-3′); *β-actin* (forward 5′-GGCACCCAGCACAATGAA-3′; reverse 5′-TAGAAGCATTTGCGGTGG-3′); *GAPDH* (forward 5′-AATCCCATCACCATCTTCC-3′; reverse 5′-CATCACGCCACAGTTTCC-3′).

### 2.7. ELISA

Cells seeded in six-well plates were treated with lumbrokinase for 48 h when they adhered to the bottom of the plate. Then, the supernatant was collected and the expression of VEGF-A and PGE2 in the supernatant was, respectively, detected by the Human VEGF ELISA Kit (D711056) and the PGE2 ELISA Kit (D751014) according to the given instructions. The kits were purchased from Sangon Biotech (Shanghai, China).

### 2.8. Flow Cytometry

Cells seeded in six-well plates were treated with different agents when the cells adhered to the plates. After 48 h, the cells were digested using EDTA-free trypsin, and the cell pellets were obtained via centrifugation at 300× *g* for 5 min twice by washing with 1 mL of PBS each time after discarding the supernatant. Then, 100 μL of 1× binding buffer was added to resuspend the pellets, and 2.5 μL FITC/AV or PI as needed was added in the dark to stain the cells. After 15–20 min, the cells were mixed with 400 μL 1× binding buffer and filtered with a 300 mesh filter to prevent machine blockage. Finally, the samples were tested immediately by BD Accuri C6 Plus Flow Cytometer (San Jose, CA, USA) or BD FACS Calibur Flow Cytometer (San Jose, CA, USA).

### 2.9. Wound Healing Assay

Cells seeded in six-well plates were gently streaked using a plastic pipette tip (200 μL) when they were grown to confluence. The non-adherent cells were washed off using PBS, and then the medium containing 1% serum and different concentrations of lumbrokinase were added. Finally, scratches were photographed at different treatment time points.

### 2.10. Transwell Assay

The matrigel was thawed overnight at 4 °C, and diluted with a serum-free medium at 1:5. Next, 70 μL of the diluted matrigel was absorbed vertically into the chamber to avoid bubbles and solidified in a 37 °C incubator for 1h. The cells were resuspended in a drug-containing medium to a concentration of 50,000 cells/mL. In total, 500 μL of medium containing 20% FBS was added to the lower chamber, and 100 μL of cell suspension was added to the upper chamber. After a quick and smooth shake, the plate was put in the cell culture incubator for 48 h. The chambers were fixed with a fixative solution for 10 min, stained with 0.1% crystal violet for 10 min, and photographed sequentially.

### 2.11. Angiogenesis Assay

On one side, a pre-cooled 96-well plate was added with 50 μL matrigel for each well and placed in a 37 °C incubator for 30 min. On the other side, HUVEC cells starved overnight were counted after digestion and resuspension, and 10,000 cells were taken and added to a 1.5 mL EP tube containing fresh conditioned medium from the supernatant of NSCLC cells treated with different agents. The cell suspension was added to the pre-coated well of the 96-well plate after repeated pipetting and mixing and the plate was placed in a 37 °C incubator for 8 h. The medium was discarded, and the cells were gently washed with PBS 3 times. Finally, 100 μL Calcein AM staining was added into each well for 30 min. After discarding the liquid and washing with PBS gently 3 times, the pictures were taken.

### 2.12. Immunofluorescence Assay

The sterile 24 mm × 24 mm slides were rinsed with PBS 3–4 times and placed in the well of a six-well plate. Then, 2 mL of agent-containing medium with 10,000 cells was added to the well. The plate was shaken well and placed in the cell culture incubator for 48 h. After being washed with PBS 3 times at room temperature, the cells on the slides were fixed with 4% paraformaldehyde for 30 min. Also, after 3 times of PBS washing, the cells on the slides were permeabilized with 0.2% Triton X-100 for 5 min. Next, the slides were washed 3 times with PBS, blocked with 10% BSA for 30 min, and incubated with the corresponding primary antibody (1% BSA dilution) at 4 °C overnight. Then, the slides were washed 3 times and incubated with fluorescent secondary antibodies at room temperature for 60 min away from light. After washing, DAPI was added to the slides to stain the cells for 4 min. With the removal of the excessive DAPI by PBS washing, the slides were sealed with anti-fluorescent quenching agent to avoid bubble formation, and finally photographed with an upright fluorescence microscope as soon as possible.

### 2.13. Pull down Assay

Biotin-labeled *VEGF*-promoter probe and *COX-2*-promoter probe, which correspond to the *VEGF* promoter sequence (−1157 to −1667) and the *COX-2* promoter sequence (−30 to −891) were synthesized by PCR using the TransStart FastPfu Fly DNA Polymerase Kit (purchased from TransGen Biotech, Beijing, China) with the biotin-labeled primers. The sequences of primers used were *VEGF* promoter (sense, 5′-AGCTGGCCTACAGAVCGTT-3′; antisense, 5′-CCCTATTTCTGACCTCCCAA-3′); *COX-2* promoter (sense, 5′-GGCCATCGCCGCTTCCTTTG-3′; antisense, 5′-AAGACTGAAAACCAAGCCCA-3′).

A total of 400 μg of the collected nuclear proteins were mixed with 3 μg of biotin-labeled *VEGF*/*COX-2*-promoter probe and 50 μL of streptavidin agarose beads in 500 μL PBSI buffer for spinning overnight at 4 °C. The beads were centrifugated at 600× *g* for 1 min, washed with PBSI twice, and then resuspended with 2× loading buffer and heated in a metal bath at 100 °C for 8 min. Finally, the samples were centrifuged at 14,000× *g* for 1 min, and the supernatant was analyzed by Western blot.

### 2.14. Chromatin Immunoprecipitation (ChIP) Assay

Cells were fixed with 1% formaldehyde, followed by 0.125 M glycine treatment to terminate the crosslinking. Then, the cells were collected with a cell scraper and the chromatin fragmentation was performed by centrifugation and ultrasonication to break DNA into 200–500 bp fragments. A portion of the total cell lysate was used as input control. The remaining lysate was immunoprecipitated with specific primary antibodies or nonspecific IgG and protein A/G agarose beads. The immunoprecipitated DNA was used as a template to amplify *VEGF* or *COX-2* promoter fragments by performing PCR. The sequences of the primers used were *VEGF* promoter (sense, 5′-AGCTGGCCTACAGACGTT-3′; antisense, 5′-ACCAAGTTTGTGGAGCTGAGAA); *COX2* promoter (sense, 5′-ATGTCAGCCTTTCTTAACCT-3′; antisense, 5′-GCAGCTTCCTGGGTTTCCGA-3′). PCR products were separated via electrophoresis on the 1% agarose gel and observed by GoldView staining.

### 2.15. Dual-Luciferase Reporter Assay

Cells transfected with sea kidney luciferase plasmids and *VEGF* promoter-driven luciferase plasmids (purchased from Youbao Biotechnology Co., Ltd., Changsha, China) or sea kidney luciferase plasmids and *COX-2* promoter-driven luciferase plasmids (constructed by our own lab) for 20 h were treated with different concentrations of lumbrokinase for another 24 h. Then, the cells were collected and lysed, and the expressions of luciferase were tested according to the protocols of Promega’s Dual-Luciferase Reporter Assay System kit (E1910, Madison, WI, USA).

### 2.16. Animal Experiments

All animal maintenance and operational procedures were carried out in accordance with the animal study protocol approved by the Animal Care and Ethics Committee of Dalian Medical University with the ethical approval number AEE17033. Male 4–6 weeks old BALB/c nude mice were purchased from Vitalstar Biotechnology Co., Ltd., Beijing, China. The animal study was mainly divided into two parts. (1) Cell-derived xenograft model: 50 nude mice were inoculated with H460 cells (1 × 10^6^ cells for each mouse) in the armpits after three to five days of adaptive feeding, but only 40 nude mice were successfully inoculated with 80% of the success rate of tumor development. When the tumor volume reached 50 mm^3^, they were randomized to 8 groups with 5 for each group to receive different treatments. The groups were as follows: (1) control group;(2) EFE group; (3) Tax group; (4) Tax + EFE group; (5) Vin group; (6) Vin + EFE group; (7) Beva group; (8) Beva + EFE group. Vincristine (5 mg/kg) or paclitaxel (2 mg/kg) was given by intraperitoneal injection twice a week, bevacizumab was given (5 mg/kg) by intraperitoneal injection three times a week, and lumbrokinase (200 mg/kg) was delivered via gastric gavage every day. The tumor diameter was recorded every two days during treatment using a vernier caliper, and the volume of the tumor was calculated according to the formula “V = (length × width^2^)/2”. After two weeks of treatment, the periocular blood of nude mice was collected from eye sockets and analyzed for creatinine and urea nitrogen using the kits purchased from Nanjing Jiancheng Bioengineering Institute, China. Then, the nude mice were killed via cervical vertebrae removal, and the tumors were removed. Partial tumor tissues were frozen in liquid nitrogen for Western blot analysis, and partial were stored in 4% paraformaldehyde for immunohistochemical analysis. (2) Matrigel plugs assay: A549 cells were digested, resuspended in an FBS-free medium, and counted. The 10^6^ cells were mixed with 200 μg/mL bevacizumab or 200 μg/mL lumbrokinase or their combination in diluted matrigel, and then 500 μL matrigel mixture was quickly injected into the subcutaneous back of nude mice. Seven days later, the nude mice were killed via cervical vertebrae removal, the formed plugs were removed, and photographs were taken. One part was used to determine hemoglobin content and the other was used for immunohistochemical analysis.

### 2.17. Immunohistochemistry Staining

Paraffin sections were dewaxed and hydrated for antigen retrieval. After that, a general two-step detection kit (PV-9000, ZSGB-Bio, Beijing, China) was used. After DAB color rendering, hematoxylin counterstaining, dehydration, transparency, and mounting, photographs were taken using an upright microscope.

### 2.18. Statistical Analysis

To compare the statistical differences between the groups, GraphPad Prism 8 software (San Diego, CA, USA) was used for analysis. Student’s *t* test or one-way ANOVA was used for bar graph analyses. Data represent the results for assays performed from at least 3 replicates, and values are the mean ± standard deviation (SD). *p* < 0.05 was considered to be a statistically significant difference.

## 3. Results

### 3.1. Lumbrokinase Induces Cellular Apoptosis, Proliferative, and Metastatic Inhibition in NSCLC Cells

**Figure 1:**
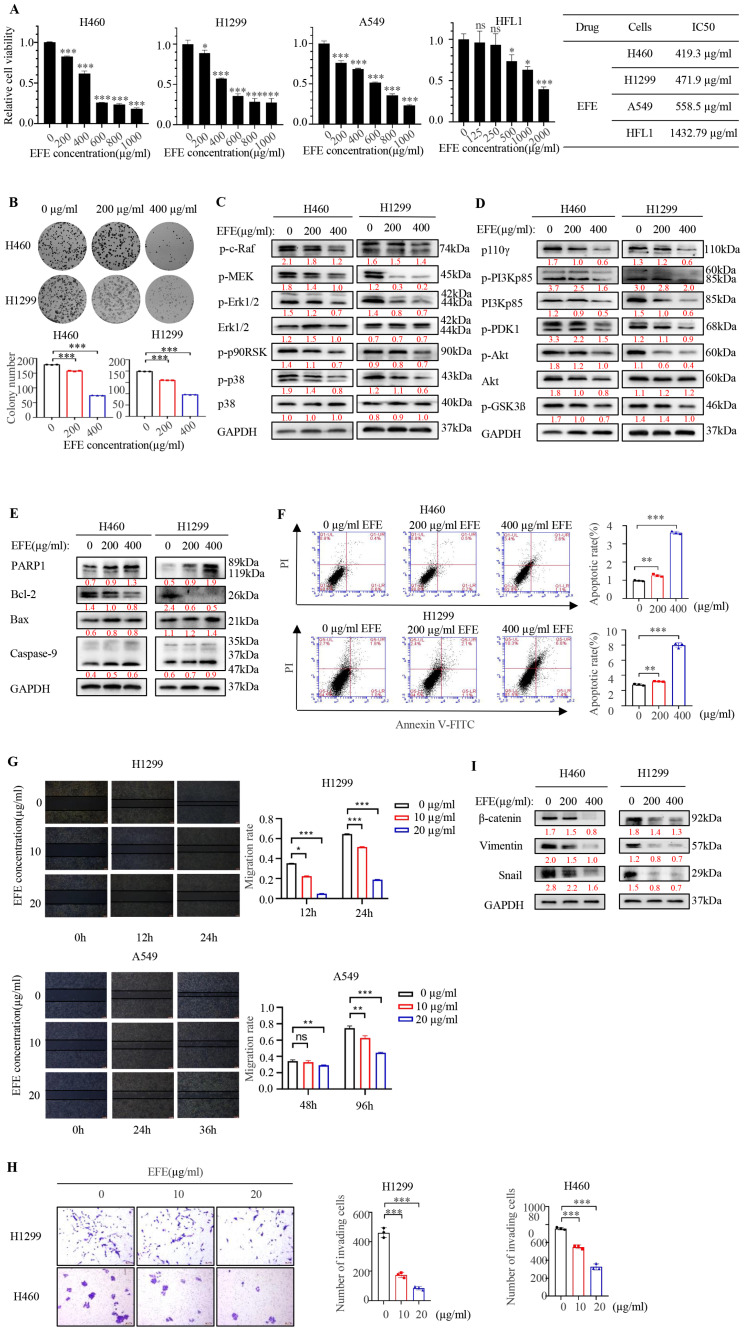
Lumbrokinase induced cellular apoptosis and inhibited proliferation and metastasis in NSCLC cells. (**A**) H460, H1299, A549, and HFL1 cells were treated with different concentrations of lumbrokinase for 48 h, and then the cell viability was tested by MTT assay. IC50 values were calculated by CVXPT32. (**B**) The colony formation assay of H460 and H1299 cells treated with lumbrokinase was detected. (**C**) H460 and H1299 cells were treated with two doses of lumbrokinase for 48 h, and the expressions of the key proteins in the MAPK/Erk signaling pathway were detected by Western blot. (**D**) The expressions of the key proteins in the PI3K/Akt signaling pathway were detected in H460 and H1299 cells with lumbrokinase treatment by Western blot. (**E**) The apoptosis-related proteins were detected by Western blot in H460 and H1299 cells treated with lumbrokinase. (**F**) FC analysis was used to detect cell apoptosis in H460 and H1299 cells treated with lumbrokinase for 48 h. The percentage of apoptotic cells was further calculated. (**G**) Wound-healing assay of H1299 and A549 cells treated with two doses of lumbrokinase for 12 and 24 h was detected (magnification 40×). The cell migration rate was calculated through the quantification of migration distance. (**H**) The invasive capacity of H460 and H1299 cells treated with two doses of lumbrokinase for 48 h was analyzed by transwell assay. The invaded cells were stained with crystal violet and the representative images were taken with an inverted microscope (magnification 40×). (**I**) The EMT-related proteins were detected by Western blot in H460 and H1299 cells treated with lumbrokinase. The data are presented as the mean ± SD. The level of significance was indicated by * *p* < 0.05, ** *p* < 0.01, and *** *p* < 0.001, and ns means no statistical significance.

We first investigated the effect of lumbrokinase on the proliferation of NSCLC cells by MTT. In H460 and H1299 cells, we found that lumbrokinase with 400 μg/mL showed the strongest inhibitory effect on NSCLC cells at 48 h (Figure S1A). Therefore, we further determined the proliferative activity of the three NSCLC cells, H460, H1299, and A549, and one normal lung cell line, HFL1, upon lumbrokinase treatment with different concentrations for 48h. Cell viability was gradually weakened with the increase in lumbrokinase’s concentration, and the IC50 value of lumbrokinase in these four different cells was calculated accordingly (Figure 1A). Compared to NSCLC cells, lumbrokinase showed comparatively low cytotoxicity to normal lung cells. Moreover, the colony formation ability of NSCLC cells was also inhibited upon lumbrokinase treatment (Figure 1B). Based on the key role played by the PI3K/AKT and MAPK/ErK signaling pathways in tumor cell growth, we next examined the effect of lumbrokinase on the expression levels of the key proteins within these two signaling pathways. After treatment with different concentrations of lumbrokinase, the phosphorylation levels of some key proteins within the MAPK/ErK signaling pathway, such as c-Raf, MEK, ErK1/2, p90RSK, and p38, decreased notably, while the expression of the total Erk and p38 was not affected (Figure 1C). Similarly, the expression levels of the key proteins within the PI3K/AKT signaling pathway, such as p110γ, p-p85, p-PDK1, p-AKT, p-GSK3β, and total AKT, decreased notably under lumbrokinase treatment (Figure 1D), indirectly indicating that lumbrokinase exerts its function in blocking the survival of NSCLC cells by inactivating these two signaling pathways. More notably, only little changes in the phosphorylation levels of some key proteins within these two signaling pathways were seen in HFL1 cells upon lumbrokinase treatment (Figure S1B,C), implying that lumbrokinase caused a significant growth inhibition on NSCLC cells but visibly weaker inhibition on normal cells.

We next analyzed the effect of lumbrokinase on the apoptosis level of NSCLC cells by detecting the expressions of apoptosis-associated proteins and flow cytometry assay. The results showed that the expression levels of cleaved PARP, Bax, and cleaved Caspase 9 increased in both tumor cells after lumbrokinase treatment, while the expression level of Bcl-2 decreased accordingly (Figure 1E). Flow cytometry analysis showed that, compared with the control group, lumbrokinase treatment remarkably promoted the apoptosis of tumor cells (Figure 1F). The above results indicate that lumbrokinase inhibits the proliferation of NSCLC cells in part by promoting cellular apoptosis.

Furthermore, we evaluated the influence of lumbrokinase on NSCLC cell metastasis. H1299 and A549 cells, which are more aggressive, were selected to execute wound healing assay and transwell assay, where the reduced concentration of lumbrokinase was applied to treat cells cultured in a medium containing 1% serum to exclude the effect of cellular proliferation on the results. Cell wound healing assay showed that a low concentration of lumbrokinase had a tendency to inhibit cell migration, and such an inhibitory effect at a high concentration was more obvious (Figure 1G). Similarly, the transwell assay showed that lumbrokinase inhibited the invasive ability of lung cancer cells, and the higher concentration caused much stronger inhibition (Figure 1H). Moreover, the expressions of the EMT-associated proteins were also influenced by lumbrokinase treatment, including β-catenin, Vimentin, and Snail (Figure 1I). These results collectively showed that lumbrokinase could inhibit the migration and invasion of NSCLC cells.

### 3.2. Lumbrokinase Synergizes with Bevacizumab in Anti-NSCLC by Targeting VEGF to Inhibit Angiogenesis

**Figure 2:**
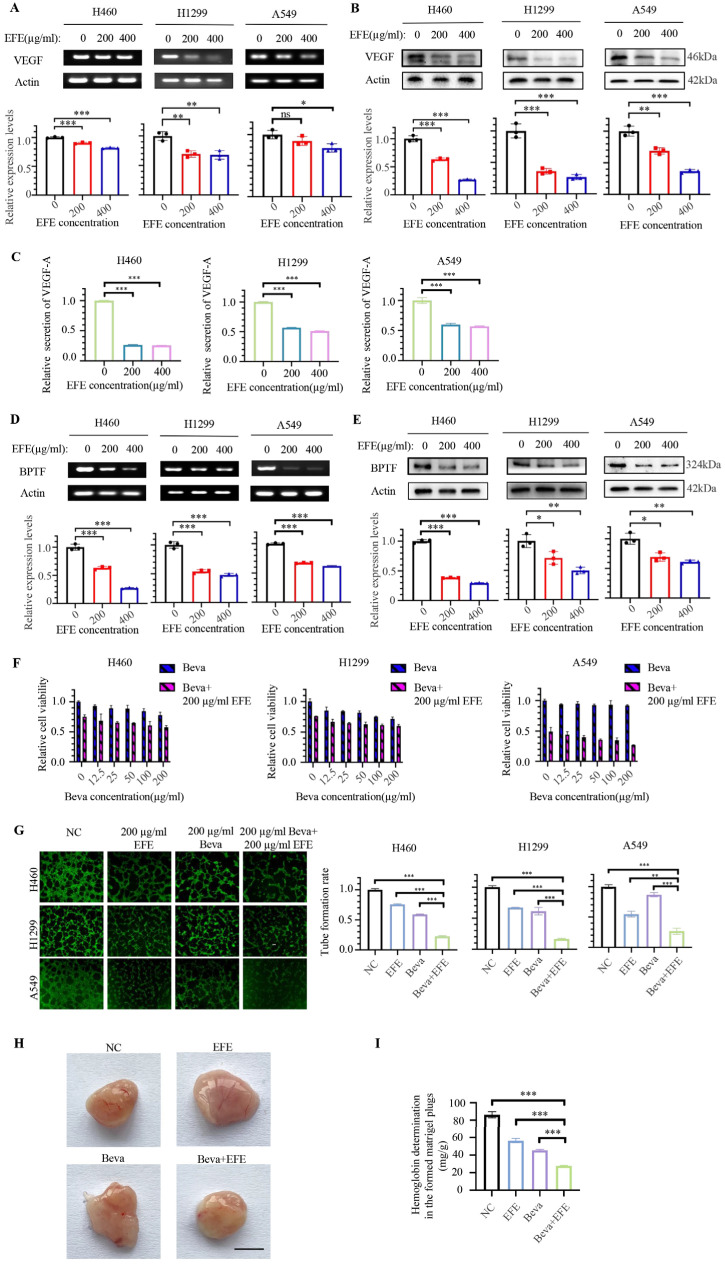
Lumbrokinase enhanced the anti-cancer efficiency of bevacizumab in NSCLC cells by targeting BPTF/VEGF signaling. (**A**) The expression of *VEGF* was analyzed at mRNA level in H460, H1299, and A549 cells after lumbrokinase treatment by RT-PCR. (**B**) The protein expression of VEGF was analyzed in H460, H1299, and A549 cells under lumbrokinase treatment by Western blot. (**C**) The level of VEGF-A in the supernatant of H460, H1299, and A549 cells treated with lumbrokinase was measured by ELISA. (**D**) The expression of *BPTF* at mRNA level in H460, H1299, and A549 cells treated with two doses of lumbrokinase for 48 h was analyzed by RT-PCR. (**E**) The protein expression of BPTF in H460, H1299, and A549 cells treated with two doses of lumbrokinase for 48 h was analyzed by Western blot. (**F**) H460, H1299, and A549 cells were treated with different concentrations of bevacizumab combined with 200 μg/mL lumbrokinase for 48 h and then tested by MTT assay. (**G**) The tube formation of HUVEC cells cultured in a medium from the supernatant of H460, H1299, and A549 cells treated with lumbrokinase and/or bevacizumab for 48 h was detected (magnification 40×). (**H**) Matrigels under different treatments were subcutaneously implanted into the mouse back and the formed matrigel plugs were taken and photographed. Scale bar = 5 mm. (**I**) The hemoglobin content of matrigel plugs was valued by the Hemoglobin Colorimetric Assay Kit. The data are presented as the mean ± SD. The level of significance was indicated by * *p* < 0.05, ** *p* < 0.01, and *** *p* < 0.001, and ns means no statistical significance.

Since lumbrokinase simultaneously inhibited PI3K/AKT and MAPK/ErK signaling pathways in NSCLC cells based on the above results and growth factors function as the common upstream molecules of these two signaling pathways, we speculate that lumbrokinase is highly likely to act on the upstream of these two pathways and influence the expression of some growth factors. We next analyzed the effect of lumbrokinase on growth factor expression in NSCLC by using different concentrations of lumbrokinase to treat H460, H1299, and A549 cells for 48 h. The expression and secretion of VEGF were detected by RT-PCR, Western blot, and ELISA assay. The results showed that the expression of *VEGF* at the mRNA level and protein level was remarkably inhibited (Figure 2A,B) and the secretion in supernatant was reduced upon lumbrokinase treatment (Figure 2C). Combined with the previous reports that the expression of VEGF is regulated by BPTF in NSCLC and BPTF functions as an oncogene to promote NSCLC progression, we next analyzed the effect of lumbrokinase on BPTF expression in NSCLC cells. We found that lumbrokinase treatment also down-regulated the expression of *BPTF* at the mRNA and protein levels (Figure 2D,E), suggesting that, most likely, lumbrokinase inactivates PI3K/AKT and MAPK/ErK signaling pathways in NSCLC cells by targeting BPTF/VEGF pathway.

Considering that Bevacizumab, an approved anti-angiogenic antibody, works by binding to VEGF and preventing it from interacting with endothelial cell surface receptors, thereby inhibiting tumor angiogenesis [34], and that lumbrokinase was found to inhibit the expression and secretion of VEGF, we hypothesized the synergy of lumbrokinase with bevacizumab in anti-tumorigenesis. We first tested the effects of bevacizumab alone or in combination with lumbrokinase on the proliferative activity of NSCLC cells. MTT assay showed bevacizumab itself only caused a weak inhibitory effect on the proliferation of NSCLC cells, while the proliferative inhibition upon the combinational treatment was mainly mediated by the addition of lumbrokinase (Figure 2F). Given that the pharmacological effect of bevacizumab is to inhibit tumor angiogenesis, we further analyzed the synergy of lumbrokinase with bevacizumab in anti-angiogenesis. Compared with lumbrokinase or bevacizumab treatment alone, the lumbrokinase combined with the bevacizumab group showed a much better inhibitory effect on angiogenesis in HUVEC cells (Figure 2G). In agreement with this, the in vivo experimental results of Matrigel Plugs referenced Langenfeld’s experimental methods [35] and showed that both lumbrokinase and bevacizumab itself inhibited tumor angiogenesis, but such inhibitory effect was more pronounced when the two agents were combined (Figure 2H). Moreover, the same conclusion was reached from the results of the hemoglobin determination in the formed matrigel plugs (Figure 2I). Altogether, the above results indicated that lumbrokinase embraces the potential to enhance the anti-angiogenesis efficiency of bevacizumab in NSCLC treatment by targeting VEGF.

### 3.3. Lumbrokinase Inhibits VEGF Expression in NSCLC Cells by Alleviating the Binding of BPTF at VEGF Promoter

**Figure 3:**
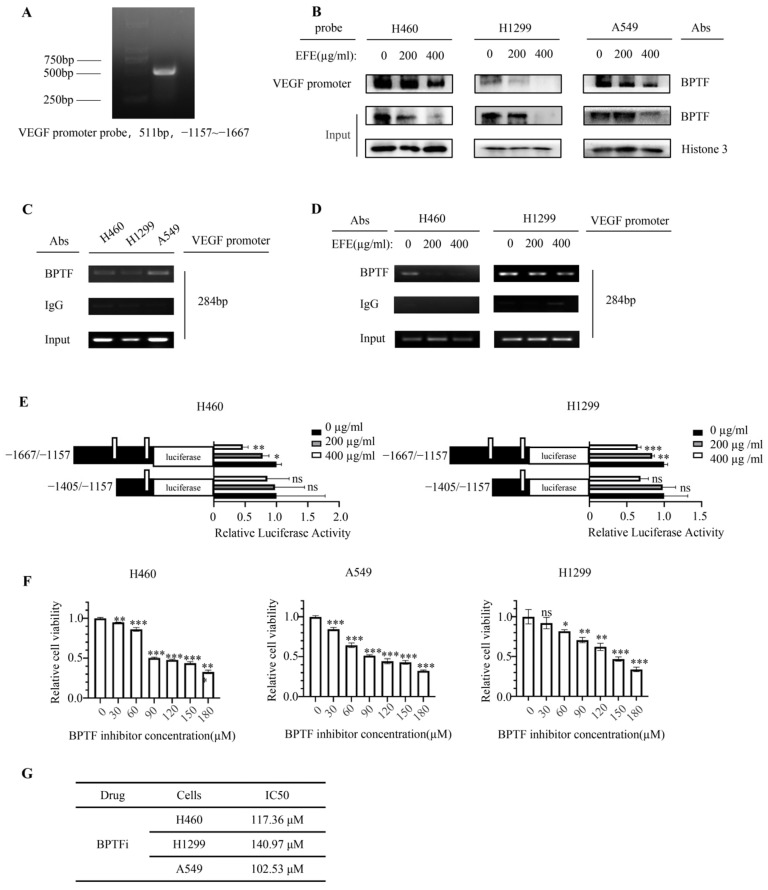

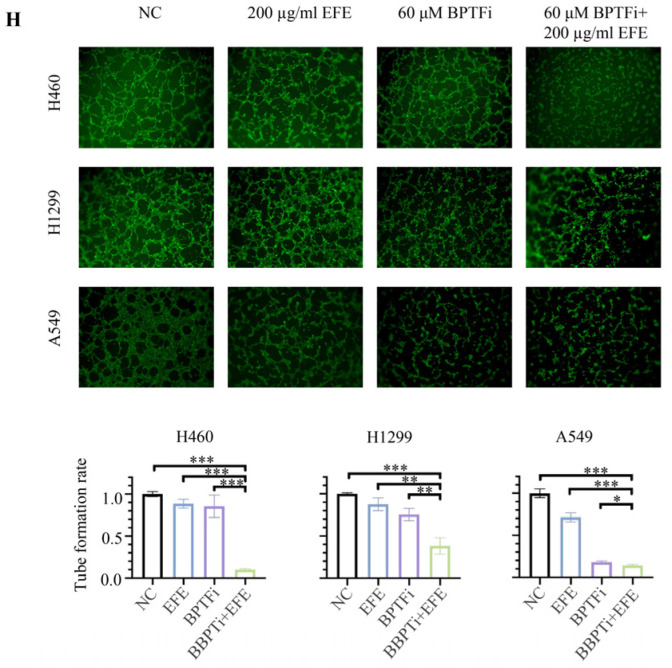
Lumbrokinase inhibited VEGF expression in NSCLC cells by alleviating the binding of BPTF at the *VEGF* promoter. (**A**) A biotinylated DNA probe of the *VEGF* promoter for the DNA pull-down experiment was synthesized by RT-PCR. (**B**) Pull-down assay using the *VEGF* promoter probe was performed in H460, H1299, and A549 cells treated with two doses of lumbrokinase, and BPTF protein was detected in the pulled-down complexes finally. (**C**) ChIP assay was performed using BPTF antibody or IgG in H460, H1299, and A549 cells, and a specific *VEGF* promoter fragment was detected finally. (**D**) ChIP assay was performed using BPTF antibody or IgG in H460 and H1299 cells treated with two doses of lumbrokinase, and a specific *VEGF* promoter fragment was detected finally. (**E**) The relative luciferase expression driven by different *VEGF* promoter fragments was detected in H460, H1299, and A549 cells after two doses of lumbrokinase treatment. (**F**) The cell viability was detected in H460, H1299, and A549 cells by MTT assay after treatment with different concentrations of BPTF inhibitors. (**G**) The IC50 values of BPTF inhibitors in three non-small lung cancer cell lines were measured by CVXPT32 software. (**H**) The tube formation of HUVEC cells cultured in a medium from the supernatant of H460, H1299, and A549 cells treated with lumbrokinase and/or BPTF inhibitors for 48 h was detected (magnification 40×). The level of significance was indicated by * *p* < 0.05, ** *p* < 0.01, and *** *p* < 0.001, and ns means no statistical significance.

In order to confirm whether lumbrokinase down-regulates VEGF expression by acting on BPTF, as our previous research reported that VEGF was regulated by BPTF in NSCLC [36], we first analyzed the binding of BPTF at the *VEGF* promoter region by DNA pull-down assay using the biotin-labeled *VEGF* promoter probe, which was equivalent to promoter region −1157 to −1667 of the *VEGF* gene (Figure 3A). The result showed that in three different NSCLC cells, BPTF bound to the *VEGF* promoter regions, and such binding decreased upon lumbrokinase treatment (Figure 3B). To further identify the specific binding site of BPTF at the *VEGF* promoter region, we segmented the *VEGF* promoter fragments used in the pull-down experiment into two parts and performed a ChIP assay to determine their interaction with BPTF, respectively. The fragment corresponding to −1384 to −1667 regions of the *VEGF* promoter was amplified in the immunoprecipitated complex (Figure 3C), but the fragment within −1157 to −1405 regions was not amplified. Moreover, the ChIP assay with the anti-BPTF antibody pulled down less *VEGF* promoter fragment (−1384 to −1667 region) in NSCLC cells with the increase in lumbrokinase treatment concentration (Figure 3D). Altogether, the above data suggested that BPTF might bind to the −1384 to −1667 regions of the *VEGF* promoter to regulate its transcription in NSCLC cells, and such binding was inhibited by lumbrokinase treatment. In agreement, we constructed luciferase-expressing plasmids driven by different *VEGF* promoter segments and performed a dual-luciferase reporter assay. The results showed that luciferase activity driven by −1157 to −1667 regions of the *VEGF* promoter was suppressed with the increase in lumbrokinase treatment concentration, but the activity driven by −1157 to −1405 regions was nearly not suppressed (Figure 3E), thereby not only suggesting the transcriptional regulation of lumbrokinase on VEGF expression but also implying that the specific regulatory sites are most likely located in the promoter region −1667–−1405 of the *VEGF* gene, which almost coincides with the binding site of BPTF at the *VEGF* promoter. Taken together, our data prove that lumbrokinase down-regulates BPTF expression, thus alleviating the binding of BPTF to the −1384 to −1667 regions of the *VEGF* promoter and then inhibiting the transcriptional activation and expression of VEGF in NSCLC cells.

In order to further explore whether the anti-angiogenic effect of lumbrokinase in NSCLC cells was related to its down-regulation on BPTF expression, the effect of lumbrokinase combined with BPTF inhibitor or not on the angiogenic capacity of HUVEC cells was investigated. BPTF inhibitor itself caused NSCLC cell growth inhibition in a concentration-dependent manner (Figure 3F). Based on the corresponding IC50 value of the BPTF inhibitor calculated according to the MTT results (Figure 3G), a 60 μM concentration of the BPTF inhibitor was selected for the angiogenesis experiment. The results showed that compared to the control group, lumbrokinase or BPTF inhibitor alone suppressed the formation of blood vessels in HUVEC cells, and the combinational use induced more pronounced inhibition (Figure 3H). Combined with our previous results, these data collectively indicate that the anti-angiogenic effect of lumbrokinase might be achieved by targeted inactivation of BPTF/VEGF signaling.

### 3.4. Lumbrokinase Sensitizes NSCLC Cells to Chemotherapeutics

**Figure 4:**
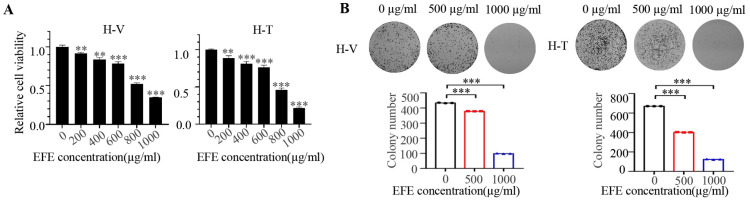

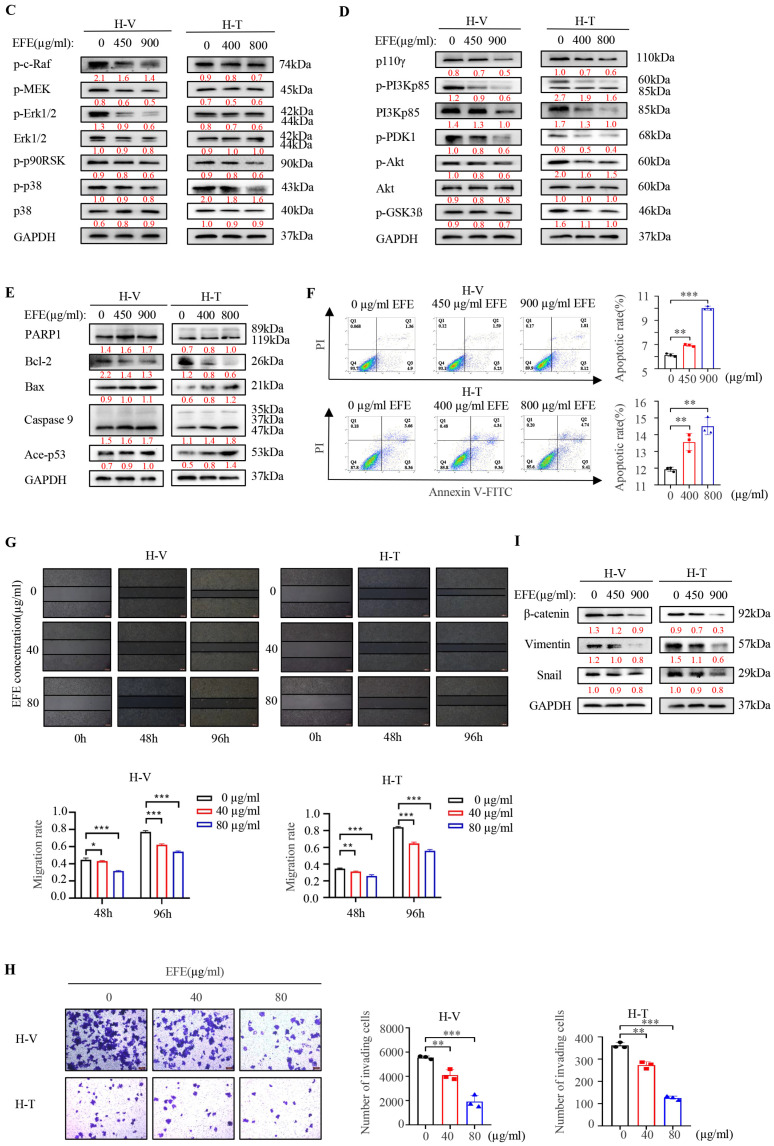
Lumbrokinase induced cellular apoptosis and inhibited proliferation and metastasis in chemo-resistant NSCLC cells. (**A**) H-T (H460 cells with paclitaxel resistance) and H-V (H460 cells with vincristine resistance) cells were treated with different concentrations of EFE for 48 h, and the cell viability was tested by MTT assay. (**B**) The colony formation assay was performed in H-T and H-V cells treated with different concentrations of EFE. (**C**) H-T and H-V cells were treated with EFE for 48 h, and the expression levels of MAPK signaling pathway-related proteins were detected by Western blot. (**D**) The expression levels of PI3K/AKT signaling pathway-related proteins in H-T and H-V cells after EFE treatment for 48 h were detected by Western blot. (**E**) The expressions of apoptosis-related proteins in H-T and H-V cells treated with EFE for 48 h were detected by Western blot. (**F**) FC analysis was used to detect cell apoptosis in H-V and H-T cells treated with EFE for 48 h. The percentage of apoptotic cells was further calculated. (**G**) Wound-healing assay of H-T and H-V cells treated with EFE was performed to detect the capacity of cell migration (magnification 40×). The cell migration rate was calculated through the quantification of migration distance. (**H**) The invasive capacity of H-T and H-V cells treated with two doses of lumbrokinase for 48 h was analyzed by transwell assay. The invaded cells were stained with crystal violet and the representative images were taken with an inverted microscope (magnification 40×). (**I**) The expressions of EMT-related proteins in H-T and H-V cells treated with EFE were detected by Western blot. The level of significance was indicated by * *p* < 0.05, ** *p* < 0.01, and *** *p* < 0.001.

To assess whether lumbrokinase could enhance the susceptibility of chemotherapy drugs, we first established vincristine- and paclitaxel-resistant NSCLC cells, namely H460-Vin (H-V) and H460-Tax (H-T). Next, we investigated the effect of lumbrokinase on the proliferation of chemo-resistant NSCLC cells. After different concentrations of lumbrokinase were added to H-V and H-T cells for 48 h, the cellular viability and proliferative ability were detected by MTT and the colony formation assay. Compared with the control group, lumbrokinase treatment obviously inhibited the proliferative potential of the two chemo-resistant cells in a concentration-dependent way (Figure 4A,B). Moreover, the expression levels of some key proteins within the MAPK/Erk and the PI3K/AKT signaling pathway, especially their phosphorylated levels, decreased considerably (Figure 4C,D). Meanwhile, we detected the cellular apoptosis change in chemo-resistant NSCLC cells under lumbrokinase treatment and found that more cellular apoptosis was induced with the increase in lumbrokinase dosage (Figure 4E,F). These results preliminarily showed that lumbrokinase could inhibit the growth of chemo-resistant NSCLC cells. In line with the study in NSCLC parental cells, we also tested the effect of lumbrokinase on the migration and invasion of chemo-resistant NSCLC cells by wound healing assay, transwell assay, and Western blot detection of EMT-related protein expression. As shown in Figure 4G–I, low-concentration of lumbrokinase could inhibit cell migration and invasion, while the inhibitory effect was more obvious upon high-concentration of lumbrokinase treatment. Combined with the previous results, these data further demonstrated that lumbrokinase exerts the function of anti-tumor cell survival not only in common NSCLC cells but also in chemo-resistant NSCLC cells.

**Figure 5:**
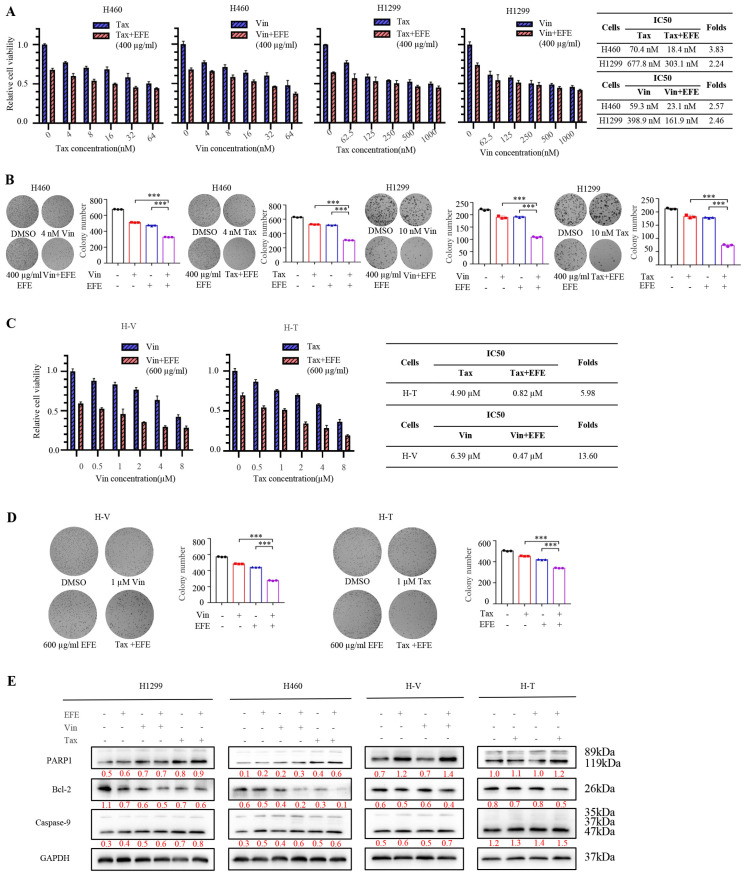

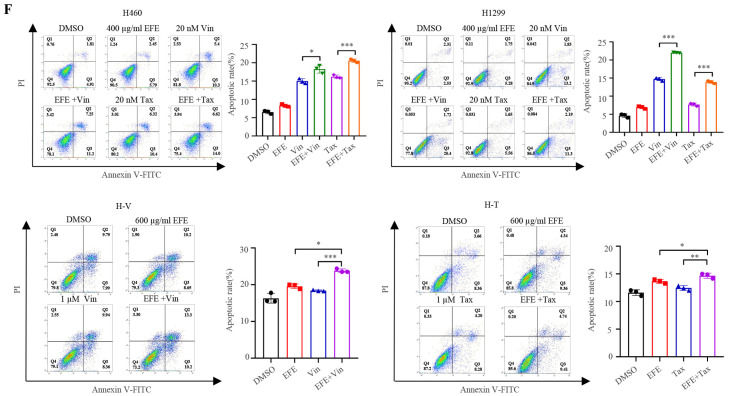
Lumbrokinase sensitized NSCLC cells to chemotherapeutics. (**A**) H460 and H1299 cells were treated, respectively, with the indicated dose of paclitaxel and vincristine alone or paclitaxel and vincristine combined with 400 μg/mL EFE for 48 h, and the cell viability was tested by MTT assay. IC50 values were calculated by CVXPT32. (**B**) The colony formation assay of H460 and H1299 cells treated, respectively, with paclitaxel and vincristine alone or paclitaxel and vincristine combined with 400 μg/mL EFE for 48 h was performed. (**C**) H460 cells with vincristine resistance (H-V) and H460 cells with paclitaxel resistance (H-T) were treated, respectively, with paclitaxel and vincristine alone or paclitaxel and vincristine combined with 600 μg/mL EFE for 48 h, and then tested by MTT assay. IC50 values were calculated by CVXPT32. (**D**) The colony formation assay of H-V and H-T cells treated, respectively, with 1uM paclitaxel and 1uM vincristine alone or paclitaxel and vincristine combined with 600 μg/mL lumbrokinase for 48 h was performed. (**E**) Western blot was used to determine the expressions of apoptosis-associated proteins in NSCLC cells under different treatments. (**F**) FC analysis was used to detect cell apoptosis in parent and chemo-resistant H460 cells treated with vincristine or paclitaxel alone or vincristine or paclitaxel combined with lumbrokinase for 48 h. The percentage of apoptotic cells was further calculated. The level of significance was indicated by * *p* < 0.05, ** *p* < 0.01, and *** *p* < 0.001.

To assess the effect of lumbrokinase on the chemotherapeutic sensitivity of NSCLC cells, we next analyzed the anti-proliferative function of chemotherapeutic drugs in combination with lumbrokinase in parent NSCLC cells. Compared with chemotherapeutic treatment alone, the combination of lumbrokinase and chemotherapeutics greatly reduced the viability of H460 and H1299 cells and ultimately reduced the IC50 values of Vin and Tax in these two cells (Figure 5A). Furthermore, beyond the two chemotherapeutics, we evaluated the IC50 values of two other commonly used chemotherapeutics, cisplatin and gemcitabine, in treating NSCLC when used in combination with lumbrokinase. As shown in Figure S2, lumbrokinase enhanced the therapeutic effect of cisplatin and gemcitabine and markedly reduced their IC50 values. Similarly, the combination of lumbrokinase and chemotherapeutics efficiently inhibited the colony formation ability of H460 and H1299 cells compared with chemotherapeutic treatment alone (Figure 5B). The same results were obtained in the two chemo-resistant NSCLC cells (Figure 5C,D). Furthermore, the cellular apoptosis analysis showed that the pro-apoptotic effect of lumbrokinase in combination with chemotherapeutics was more pronounced than that of the chemotherapeutic treatment alone (Figure 5E,F), suggesting that lumbrokinase has the potential to enhance the chemotherapeutic sensitivity of NSCLC cells, that is, to synergize with chemotherapeutics to treat NSCLC.

### 3.5. Lumbrokinase Sensitizes NSCLC Cells to Chemotherapeutics by Targeting NF-κB/COX-2 Signaling

**Figure 6:**
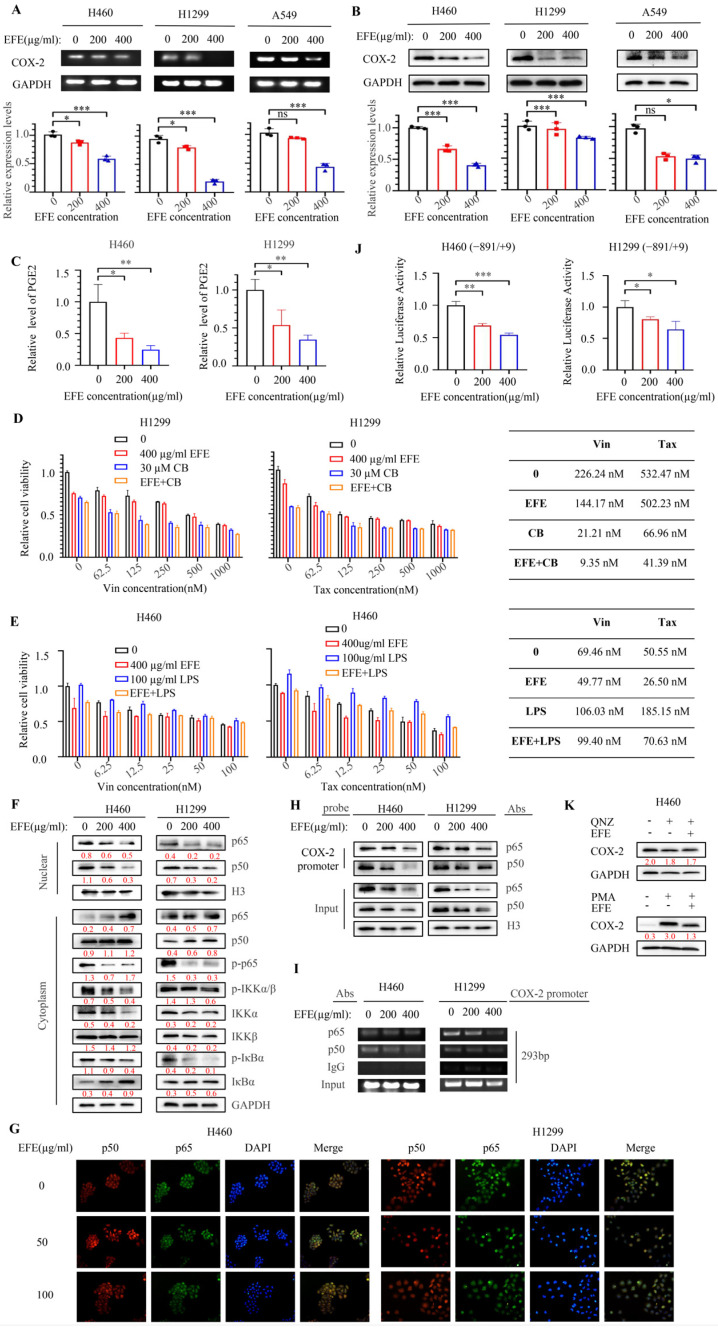
Lumbrokinase sensitized NSCLC cells to chemotherapeutics by targeting NF-κB/COX-2 signaling. (**A**) The expression level of *COX-2* in H460, H1299, and A549 cells treated with lumbrokinase for 48 h was analyzed by RT-PCR. (**B**) The expression level of COX-2 in H460, H1299, and A549 cells treated with lumbrokinase for 48 h was analyzed by Western blot. (**C**) The level of PGE2 in the supernatants of H460 and H1299 cells treated with lumbrokinase for 48 h was measured by ELISA. (**D**) The chemotherapeutic sensitivity was tested by MTT assay in H1299 cells treated with/without lumbrokinase and/or CB. (**E**) The chemotherapeutic sensitivity was tested by MTT assay in H460 cells treated with/without lumbrokinase and/or LPS. (**F**) The key proteins of the NF-κB pathway were detected in the cytoplasm or nucleus in NSCLC cells treated with different concentrations of lumbrokinase. (**G**) The distribution of p50 and p65 protein in NSCLC cells treated with different doses of lumbrokinase by immunofluorescence (magnification 200×). (**H**) Pull-down assay using a *COX2* promoter probe was performed in H460 and H1299 cells under two doses of lumbrokinase treatment, and the p50 and p65 proteins were detected. (**I**) ChIP assay was performed using p50 and p65 antibodies or IgG in H460 and H1299 cells under two doses of lumbrokinase treatment. (**J**) The relative luciferase expression driven by the *COX2* promoter in H460 and H1299 cells was detected upon lumbrokinase treatment. (**K**) The expression of COX2 in H460 cells treated with QNZ or PMA alone or its combination with lumbrokinase. The level of significance was indicated by * *p* < 0.05, ** *p* < 0.01, and *** *p* < 0.001, and ns means no statistical significance.

Given that lumbrokinase has previously been reported to inhibit the upregulation of COX-2 expression caused by cardiac deficiency, reperfusion injury [37], and COX-2/mPGES-1/PGE2 signaling axis has been reported to be involved in the self-renewal and chemotherapeutic resistance of cancer stem cells [38,39], we speculated that the enhancement of chemosensitivity mediated by lumbrokinase in NSCLC cells might also be achieved by its targeted regulation on COX-2. We first examined the changes in COX-2 expression and PGE2 secretion after lumbrokinase treatment in H460, H1299, and A549 cells. The results showed that the expression of *COX-2* in NSCLC cells was inhibited at the mRNA and protein levels upon lumbrokinase treatment (Figure 6A,B), and PGE2 secretion was also correspondingly reduced (Figure 6C). Next, we observed the influence of COX-2 activation or inhibition on the synergy of lumbrokinase with chemotherapeutics in NSCLC cells. To select a suitable concentration, we analyzed the effect of COX-2 inhibitor celecoxib and the COX-2 activator LPS on cell viability in H460 and H1299 cells and found that celecoxib inhibited NSCLC cell viability, while LPS treatment caused the reverse trend (Figure S3A,B). Not only that, but the rescue assay also showed that celecoxib treatment blocked the anti-proliferative function of lumbrokinase (Figure S3C), while LPS treatment rescued the inhibition of cell proliferation caused by lumbrokinase (Figure S3D). In the following chemo-sensitivity assay, we found celecoxib treatment similarly enhanced the sensitivity of NSCLC cells to chemotherapeutics, and its combination with lumbrokinase produced the best synergy with chemotherapeutics (Figure 6D), and the induction of LPS reversed such synergy (Figure 6E), indicating that lumbrokinase enhanced the chemotherapy sensitivity of NSCLC cells by targeting COX-2.

In order to further explore the underlying molecular mechanism of lumbrokinase in regulating COX-2 expression, we detected the expressions and distributions of some key proteins within the NF-κB signaling pathway in NSCLC cells upon lumbrokinase treatment based on the known regulation of NF-κB signaling on COX-2 expression [40,41]. Lumbrokinase application suppressed the activation of the NF-κB signaling pathway and led to the reduced translocation of p50 and p65 from the cytoplasm to the nucleus (Figure 6F,G). Furthermore, we analyzed the binding of p50 and p65 at the *COX-2* promoter region upon lumbrokinase treatment in NSCLC cells. A biotin-labeled *COX-2* promoter probe corresponding to −891 to −30 fragment within the *COX-2* promoter was designed (Figure S3E), and a DNA pull-down assay was performed. The results showed that the binding of p50 and p65 at the *COX-2* promoter probe was gradually weakened with the increase in lumbrokinase treatment concentration (Figure 6H). ChIP assay obtained the same results, where a relatively short region of the *COX-2* promoter segment from −488 to −196 was analyzed (Figure 6I). In addition, the dual-luciferase reporter assay was performed, and lumbrokinase application inhibited *COX-2* promoter-driven luciferase expression (Figure 6J). When the NF-κB inhibitor QNZ was used, COX-2 expression was significantly decreased, which is consistent with the result of Vinay Shukla’s study [42], while COX-2 expression was virtually unchanged after superimposing lumbrokinase to QNZ. In contrast, the NF-κB activator PMA treatment greatly increased the expression of COX-2, and the superimposed lumbrokinase reversed the increase caused by PMA (Figure 6K). Taken together, these data prove that the ability of lumbrokinase to enhance the chemotherapy sensitivity of NSCLC cells was at least partially achieved by inactivating the NF-κB signaling pathway, thus alleviating the binding of p50/p65 at *COX-2* promoter and subsequent COX-2 expression.

### 3.6. Lumbrokinase Inhibits NSCLC Survival In Vivo via Combination with Bevacizumab or Chemotherapeutics

**Figure 7:**
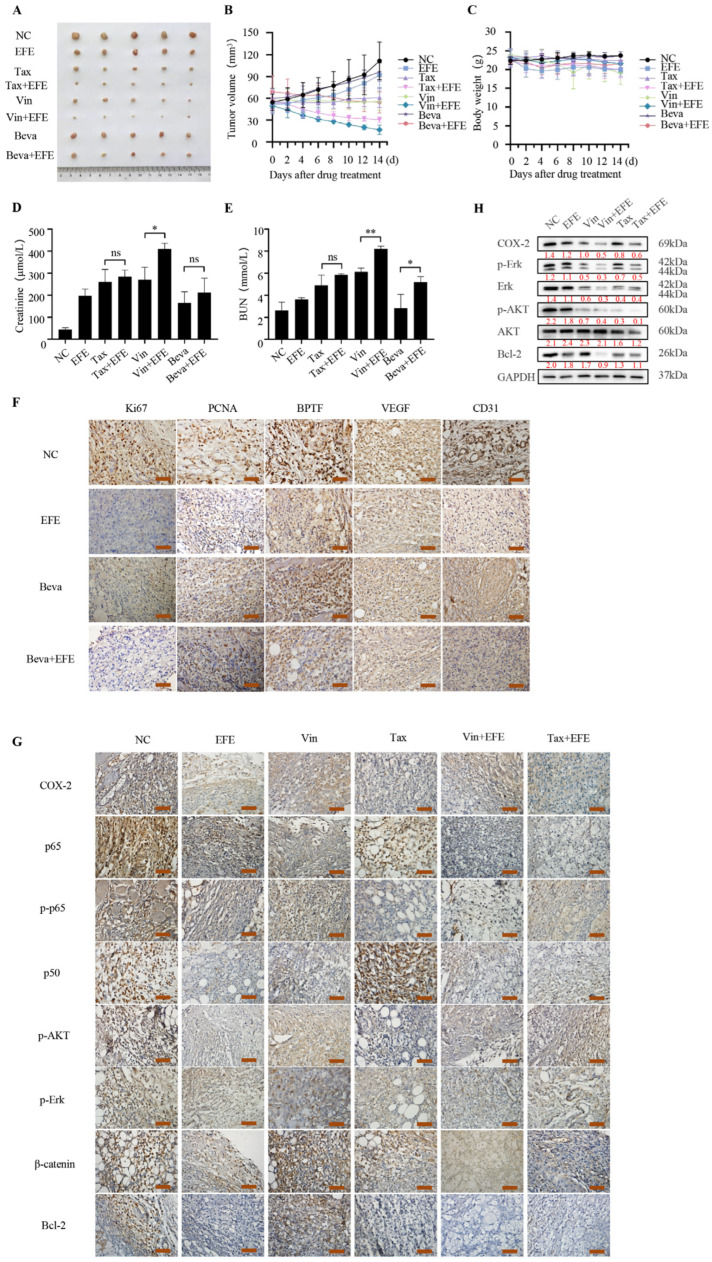
Lumbrokinase enhanced the anti-tumor effect of chemotherapy agents or bevacizumab in the xenograft of human NSCLC cells in mice. (**A**) Images of xenograft tumor harvested after therapy. (**B**) Dynamic development of tumor volume during the therapy. (**C**) Dynamic development of body weight during the therapy. (**D**) Serum creatinine levels of mice in each group after therapy. (**E**) Blood Urea Nitrogen (BUN) level of mice in each group after therapy. (**F**) Immunohistochemical staining assay shows the expression levels of BPTF, VEGF, Ki67, PCNA, and CD31 in tissue sections. The representative images were taken by an upright microscope. Scale bar = 50 μm. (**G**) Immunohistochemical staining assay shows the expressions of COX2, p-p65, p-Akt, p-Erk, and Bcl-2 in tissue sections. The representative images were taken by an upright microscope. Scale bar = 50 μm. (**H**) The expression levels of COX-2, p-Erk, Erk, p-Akt, Akt, and Bcl-2 in tumor tissue lysates were detected by Western blot. The level of significance was indicated by * *p* < 0.05, ** *p* < 0.01, and ns means no statistical significance.

Our previous results showed that lumbrokinase synergized with bevacizumab or chemotherapeutics in inhibiting the growth of NSCLC cells in vitro. In order to further verify this synergy in vivo, we constructed a subcutaneous xenograft tumor model of NSCLC cells in mice by inoculating 1 × 10^7^ H460 cells in the armpits of nude mice. The mice were randomly grouped when the formed tumor grew to 50 mm^3^ and began to receive treatments for 2 weeks. As shown in Figure 7A,B, the tumor volume of the mice receiving monotherapy was decreased compared with the control group, and the combinational therapy with lumbrokinase and bevacizumab, lumbrokinase and vincristine, or paclitaxel resulted in better tumor growth inhibition than the single agent treatment. In addition, compared to monotherapy, lumbrokinase combined with bevacizumab, vincristine, or paclitaxel administration had little influence on the body weight of mice, and only caused a slight elevation of creatinine and urea nitrogen levels. Moreover, no acute or delayed side effects were seen in mice receiving the combinational treatment (Figure 7C–E).

Furthermore, the expressions of some key proteins in tumor tissues among different treatment groups were detected by immunohistochemical staining and Western blot assay. As shown in Figure 7F, the expression of Ki67 and PCNA in the combined administration group of lumbrokinase and bevacizumab was markedly reduced compared with the control group, lumbrokinase treatment group, or bevacizumab treatment group. In addition, the expression of BPTF and VEGF in the combined treatment group was also reduced, which was consistent with the results of in vitro experiments. The expression of CD31, a sensitive and specific marker for vascular differentiation, was also down-regulated upon treatment, especially the combinational treatment (Figure 7F), indicating that the combination administration of lumbrokinase and bevacizumab could produce a more pronounced anti-tumor growth effect in the xenografts of NSCLC cells by inhibiting tumor angiogenesis and development.

At the same time, immunohistochemical staining of the tumor tissues in different groups treated with lumbrokinase and/or chemotherapeutics showed that compared with the control group, the expressions of COX-2, phosphorylated AKT and Erk, β-catenin, and Bcl-2, and the location of p50 and p65 in the nucleus, were all decreased upon treatment, and this reduced trend was more pronounced in the combined group (Figure 7G). Similarly, Western blot assay of tumor tissues in different groups also showed that the expressions of COX-2, p-Erk, Erk, p-AKT, and Bcl-2 were markedly decreased upon the combinational treatment of lumbrokinase with vincristine or paclitaxel, compared to the single agent treatment, but the expression of the total AKT remained nearly unchanged (Figure 7H). These results proved again that lumbrokinase synergizes with bevacizumab or chemotherapeutics in antagonism to NSCLC progression by inactivating BPTF/VEGF and NF-κB/COX-2 signaling to inhibit the formation of tumor blood vessels and enhance the chemosensitivity of tumor cells.

## 4. Discussion

The high aggressiveness, metastasis, and tolerance to chemotherapy/radiotherapy severely limited the averaged survival time of lung cancer patients after diagnosis [43], and it is imperative to propose new treatment strategies, especially the new potential combinational therapeutic strategies, to improve the outcome of lung cancer patients. Lumbrokinase, extracted from *earthworms*, is a complex enzyme with fibrinolytic ability [44]. It plays an essential role in achieving anticoagulation by inhibiting platelet function [45]. Although some studies have recently reported the anti-tumor effects of lumbrokinase in several cancer types, including liver cancer, gastric cancer, and breast cancer [46], its precise function in antagonizing lung cancer progression and its potential in synergizing with other treatment forms to co-treat lung cancer are still largely unknown. Here, we explored and confirmed lumbrokinase as a potential anti-cancer agent used alone or in combination with other treatment forms, including anti-angiogenesis therapy and chemotherapy, in lung cancer development, especially in NSCLC development. Mechanistically, we found that lumbrokinase, on the one hand, not only simultaneously inhibited PI3K/AKT and MAPK/ErK signaling pathways in NSCLC cells but also down-regulated the common upstream regulatory molecule of these two pathways, VEGF, by targeting BPTF and reduced the binding of BPTF at the *VEGF* promoter region, which in turn impaired the transcription of *VEGF* and sensitized NSCLC cells to bevacizumab therapy; on the other hand, inactivated NF-κB signaling pathway reduced the translocation of p50/p65 from the cytoplasm into nucleus and alleviated their binding at *COX-2* promoter, which in turn repressed COX-2 expression and sensitized NSCLC cells to chemotherapy.

Given that angiogenesis plays a key role in the development and metastasis of malignant tumors and the VEGF/VEGFR signaling pathway is exceedingly vital in angiogenesis, the targeted inhibition of this pathway has arisen as one of the important anti-tumor angiogenesis therapeutic strategies [47]. Our study found the down-regulation of *VEGF* at both protein and mRNA levels in NSCLC cells upon lumbrokinase treatment, suggesting the possible role of lumbrokinase in inhibiting tumor angiogenesis, thereby restraining the progression of NSCLC, which has been proven by our in vitro and in vivo study. Bevacizumab, approved by the FDA in 2004 and widely used in anti-cancer therapy [16], is a class of monoclonal antibodies against VEGF and is rarely used alone because of a lack of obvious efficacy, but it is mostly combined with platinum-based chemotherapeutics, which inevitably leads to a variety of adverse effects [48,49]. Therefore, we naturally hypothesized and proved the synergy of lumbrokinase with bevacizumab in anti-tumor angiogenesis and ultimately in anti-tumor progression, evidenced by the improved inhibition of angiogenesis in HUVEC cells in vitro and in Matrigel plugs assay in vivo upon the combinational treatment of bevacizumab and lumbrokinase. More notably, the application of bevacizumab in combination with lumbrokinase enhanced the anti-cancer efficacy of bevacizumab itself without increasing its toxic side effects in the xenograft model of human NSCLC cells in mice, implying the great potential of such combinational therapeutic strategy for clinical application. Besides bevacizumab targeting VEGF, anti-vascular therapy also includes some other agents, such as antirotinib, recombinant human endostatin, and apatinib, which all target the receptor of VEGF or VEGFR [50]. The possibility and potential of lumbrokinase in combination with any of these agents to improve its anti-tumor angiogenesis efficacy are in need of further exploration.

More interestingly, we found that lumbrokinase down-regulated VEGF expression via the targeted suppression of BPTF. Considering the important role of BPTF in tumor progression as a ubiquitously expressed ATP-dependent chromatin-remodeling factor according to the reports in recent years [51,52,53,54,55], our study not only revealed the anti-tumor effect of lumbrokinase, which is at least partially realized by targeting BPTF, but also uncovered the underlying molecular mechanisms of lumbrokinase in regulating VEGF expression. DNA pull-down experiment and ChIP assay verified the binding of BPTF at the specific *VEGF* promoter region in NSCLC cells, and this binding was found to be weakened upon lumbrokinase treatment. Combined with the dual-luciferase experiment, where lumbrokinase treatment inhibited *VEGF* promoter-driven luciferase expression and the down-regulation of BPTF caused by lumbrokinase, our current results collectively proved that lumbrokinase suppresses the transcriptional activity of *VEGF* and its subsequent expression by targeting BPTF, illustrating the crucial significance of BPTF in this regulation. In line with this, BPTF inhibitor alone showed a certain anti-angiogenesis effect, while lumbrokinase combined with BPTF inhibitor showed a more significant effect on inhibiting angiogenesis in vitro, suggesting that the targeted inhibition of BPTF might become a new option for anti-tumor angiogenesis strategies. Combined with the existing reports, our study further enriched the anti-tumor function of BPTF, especially its indispensable role in the anti-tumor angiogenesis function mediated by lumbrokinase.

Although chemotherapy is still the main form among the first-line treatment strategies for advanced NSCLC, the inevitable toxic side effects and the acquired resistance to these therapies remain significant clinical challenges [56]. In our study, lumbrokinase was found to enhance the efficacy of chemotherapeutics and to overcome their resistance to some extent via the targeted inactivation of the NF-κB/COX-2 signaling pathway. Considering the previous reports that the overexpression of COX-2 promotes cancer stemness and chemotherapy resistance [57], while its inhibitor celecoxib causes the reverse effects [58], and that lumbrokinase can inhibit the enhancement of myocardial ischemia–reperfusion-induced expressions of COX-2 [37], we hypothesized and confirmed the critical role of COX-2 in the synergy of lumbrokinase with therapeutics in NSCLC treatment. As we speculated, the use of COX-2 inhibitor celecoxib greatly increased the inhibitory effect of lumbrokinase on the proliferation of NSCLC cells and further reduced the IC50 value of the chemotherapeutics, while LPS induction reversed this sensitization. Not only that, we also found that the inhibition of COX-2 expression upon lumbrokinase treatment was achieved by inactivating the NF-κB pathway, which is evidenced by the results that, under the action of lumbrokinase, the phosphorylation of the key proteins within the NF-κB pathway were blocked, the translocation into the nucleus of p50/p65 was decreased, the binding of p50/p65 at *COX-2* promoter regions was alleviated, and the transcriptional activity of *COX-2* was inhibited. In addition, the use of lumbrokinase itself down-regulates the increase in COX-2 expression due to the action of the NF-κB activator PMA, whereas when lumbrokinase was used in combination with the NF-κB inhibitor QNZ, the expression of COX-2 was almost the same as the inhibitor treatment alone, indicating that the inhibition of COX-2 expression mediated by lumbrokinase was almost entirely achieved by inactivating NF-κB. It has been reported that COX-2 inhibitors can enhance the sensitivity of cancer cells to a variety of chemotherapeutics, such as cisplatin, oxaliplatin, and 5-FU [59,60], not just to the chemotherapeutics we are currently studying here. Does lumbrokinase have a synergistic anti-tumor effect with other chemotherapeutics in NSCLC therapy? If so, does it also work by inhibiting COX-2 expression, or through some other responsive mechanisms? All these issues similarly deserve further exploration.

**Figure 8:**
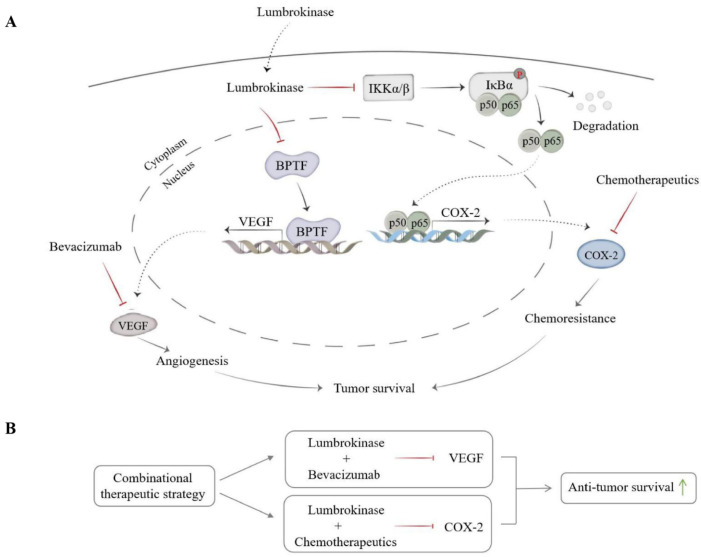
Schematic diagram of the mechanism by which lumbrokinase synergizes with bevacizumab or chemotherapeutics in NSCLC therapy. (**A**) The schematic diagram of the molecular mechanisms by which lumbrokinase inhibits tumor survival via inactivating BPTF/VEGF and NF-κB/COX-2 signaling, respectively. (**B**) The schematic diagram of combination therapy strategies by applying lumbrokinase with bevacizumab or chemotherapeutics in anti-NSCLC.

In summary, our study explored and confirmed the anti-tumor effect of lumbrokinase in NSCLC, especially its synergy with bevacizumab or chemotherapeutics in NSCLC therapy. Not only that, we also uncovered the molecular mechanism underlying such synergy mediated by lumbrokinase via in vitro and in vivo studies (Figure 8A,B). Lumbrokinase down-regulated the expression of BPTF and reduced the binding of BPTF at the *VEGF* promoter region, which in turn inhibited the transcription of *VEGF* and ultimately restrained tumor vascular prosperity and sensitized NSCLC to bevacizumab treatment. Meanwhile, lumbrokinase inactivated NF-κB signaling, reduced the translocation of p50/p65 into the nucleus and their binding at the *COX-2* promoter region, and restrained the transcription of *COX-2*, thereby enhancing the anti-tumor effect of chemotherapeutics. Therefore, our current results indicate that lumbrokinase is a potential therapeutic agent, either in isolation or in combination with bevacizumab or chemotherapeutics, in NSCLC treatment, while its combination with anti-angiogenic agents or chemotherapeutics is expected to be provided as a new treatment option to improve the prognosis of patients with NSCLC.

## 5. Conclusions

In this study, we report the critical role of lumbrokinase in suppressing NSCLC survival via restraining cell proliferation, migration, and invasion, and promoting cellular apoptosis. Lumbrokinase can be used in isolation as a potential anti-NSCLC drug. Moreover, lumbrokinase was found to inhibit tumor angiogenesis and sensitize NSCLC cells to bevacizumab via the targeted down-regulation of BPTF, weakening the binding of BPTF to the *VEGF* promoter, thereby decreasing the expression of VEGF. Meanwhile, lumbrokinase enhances the therapeutic efficacy of chemotherapeutics and partly reverses the chemoresistance of NSCLC cells via the inactivation of NF-κB signaling, reducing the translocation of p50/p65 from the cytoplasm to the nucleus and their binding to the *COX-2* promoter, thereby inhibiting COX-2 expression. Therefore, the application potential of lumbrokinase in combination with bevacizumab or chemotherapeutics in the treatment of NSCLC was highlighted. Lumbrokinase is expected to be provided as a clinical adjuvant drug of bevacizumab or chemotherapeutics to improve the outcome of NSCLC patients.

## Data Availability

The raw data supporting the conclusions of this article will be made available by the corresponding author upon request.

## Supplementary Materials

The following supporting information can be downloaded at: https://www.mdpi.com/article/10.3390/biom14070741/s1, Figure S1. Lumbrokinase was cytotoxic to NSCLC cells but not to normal lung cells. Figure S2. Lumbrokinase sensitized NSCLC cells to cisplatin and gemcitabine treatment; Figure S3. Lumbrokinase sensitized NSCLC cells to chemotherapeutics by targeting NF-κB/COX-2 signaling; Figure S4. Uncropped images of Western blot membranes for Figure 1; Figure S5. Uncropped images of Western blot membranes for Figure 2; Figure S6. Uncropped images of Western blot membranes for Figure 3; Figure S7. Uncropped images of Western blot membranes for Figure 4; Figure S8. Uncropped images of Western blot membranes for Figure 5; Figure S9. Uncropped images of Western blot membranes for Figure 6; Figure S10. Uncropped images of Western blot membranes for Figure 7; Figure S11. Uncropped images of Western blot membranes for Figure S1.
