# Supplementary material for: Lumbrokinase Extracted from Earthworms Synergizes with Bevacizumab and Chemotherapeutics in Treating Non-Small Cell Lung Cancer by Targeted Inactivation of BPTF/VEGF and NF-κB/COX-2 Signaling
Source: Biomolecules. 2024 Jun 23;14(7):741. doi: 10.3390/biom14070741 (PMC11274885; doi:10.3390/biom14070741)
Supplement: Supplementary file 1 [file biomolecules-14-00741-s001.zip › biomolecules-3011215-supplementary/biomolecules-3011215-supplementary 2.pdf]

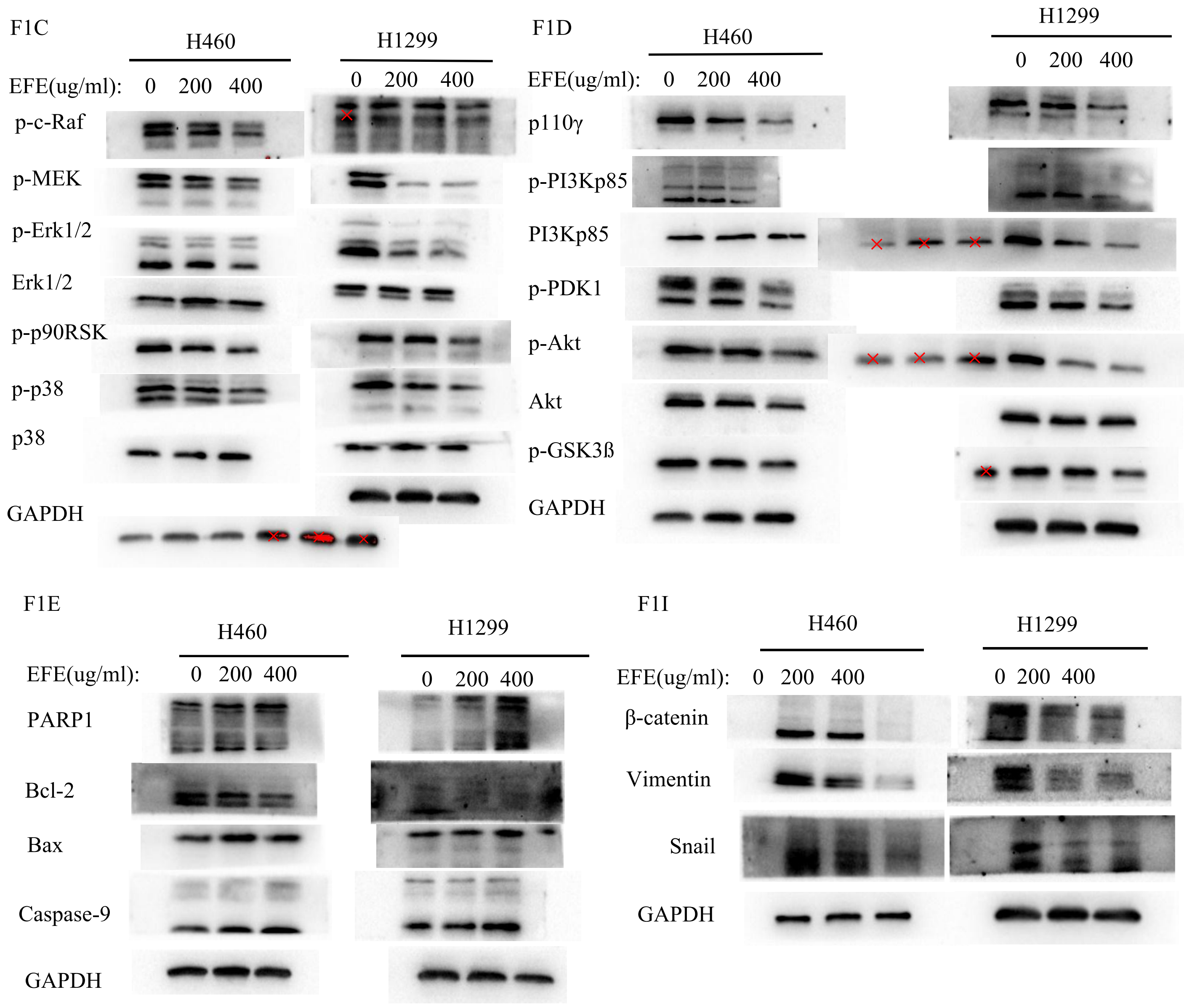


**Figure S4. Uncropped images of Western blot membranes for Figure 1.** Note that all membranes were re-probed for GAPDH, but only one GAPDH representative is shown in Figure 1. All proteins were quantified using their respective GAPDH for the corresponding membranes. Signal quantifications are provided in Figure 1.

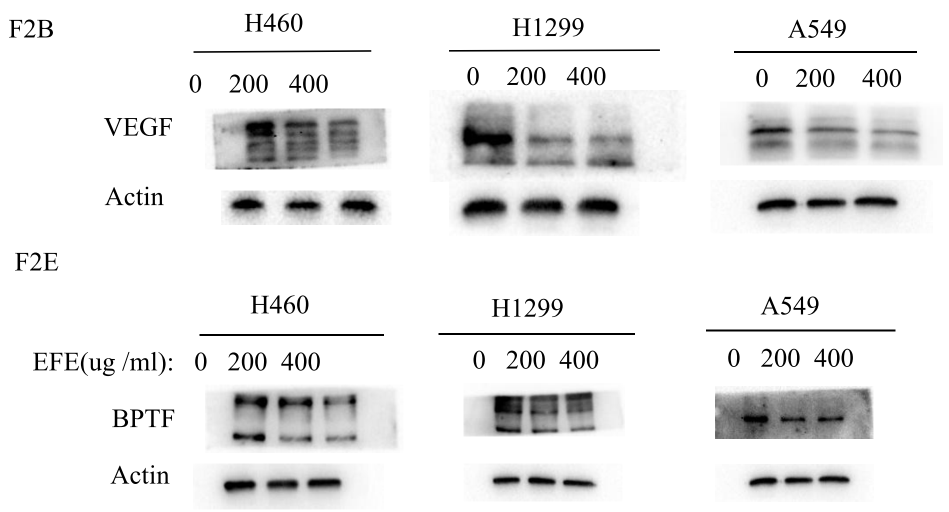


**Figure S5. Uncropped images of Western blot membranes for Figure 2.** All proteins were quantified using their respective Actin for the corresponding membranes. Signal quantifications are provided in Figure 2.

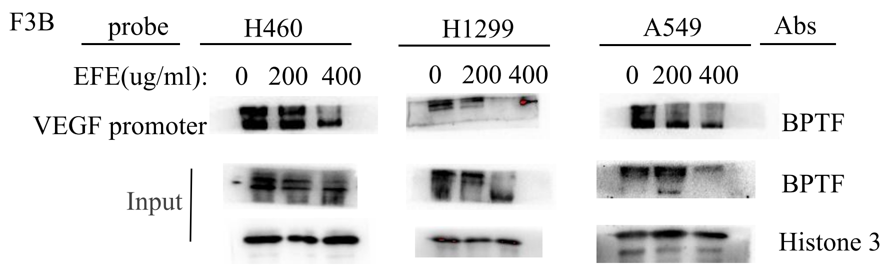


**Figure S6. Uncropped images of Western blot membranes for Figure 3.**

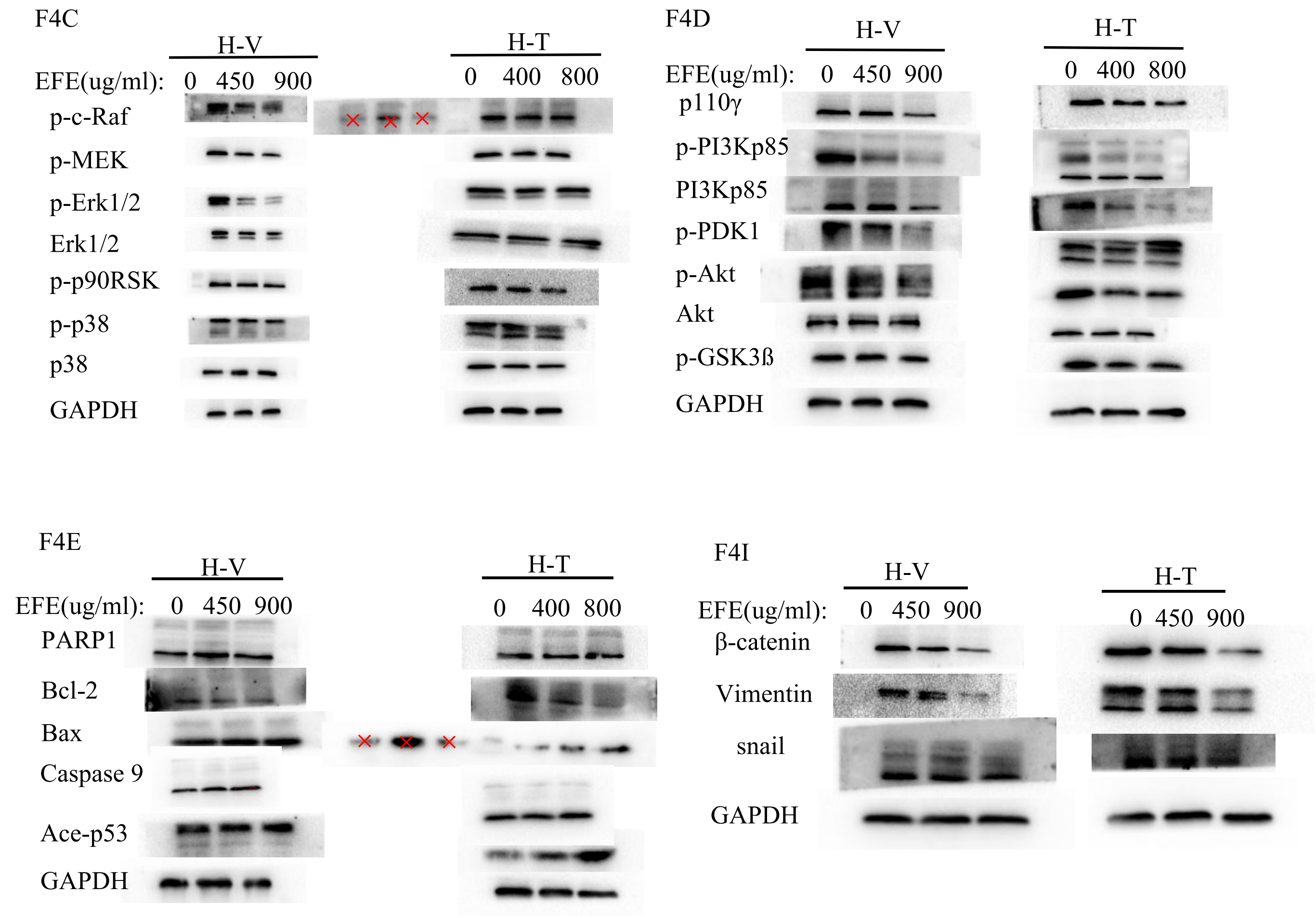


**Figure S7. Uncropped images of Western blot membranes for Figure 4.** Note that all membranes were re-probed for GAPDH, but only one GAPDH representative is shown in Figure 4. All proteins were quantified using their respective GAPDH for the corresponding membranes. Signal quantifications are provided in Figure 4.

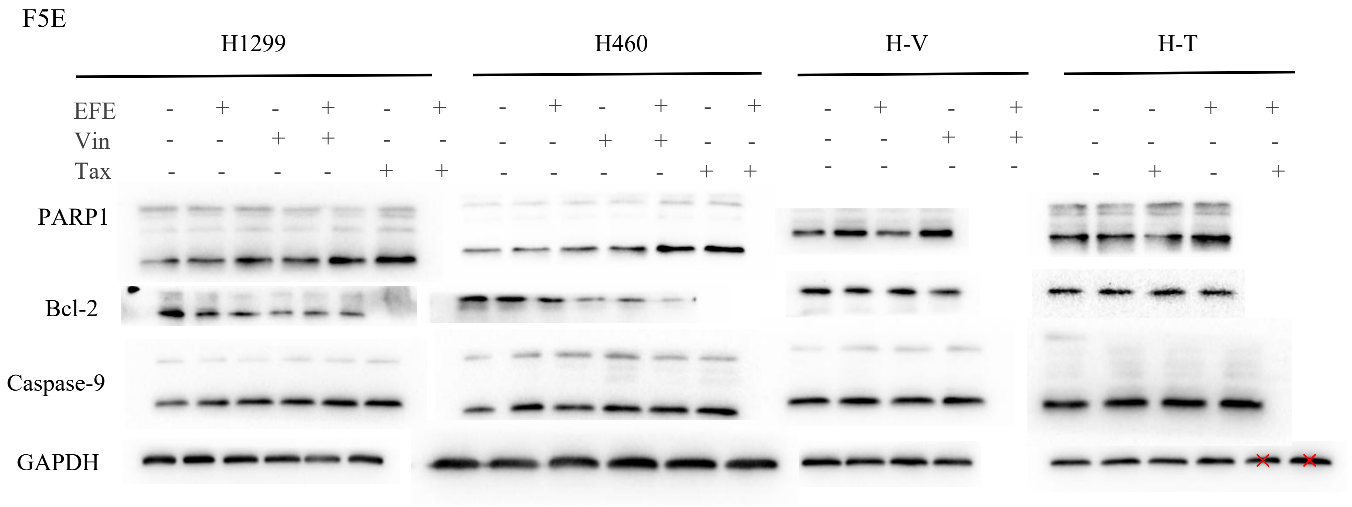


**Figure S8. Uncropped images of Western blot membranes for Figure 5.** Note that all membranes were re-probed for GAPDH, but only one GAPDH representative is shown in Figure 5. All proteins were quantified using their respective GAPDH for the corresponding membranes. Signal quantifications are provided in Figure 5.

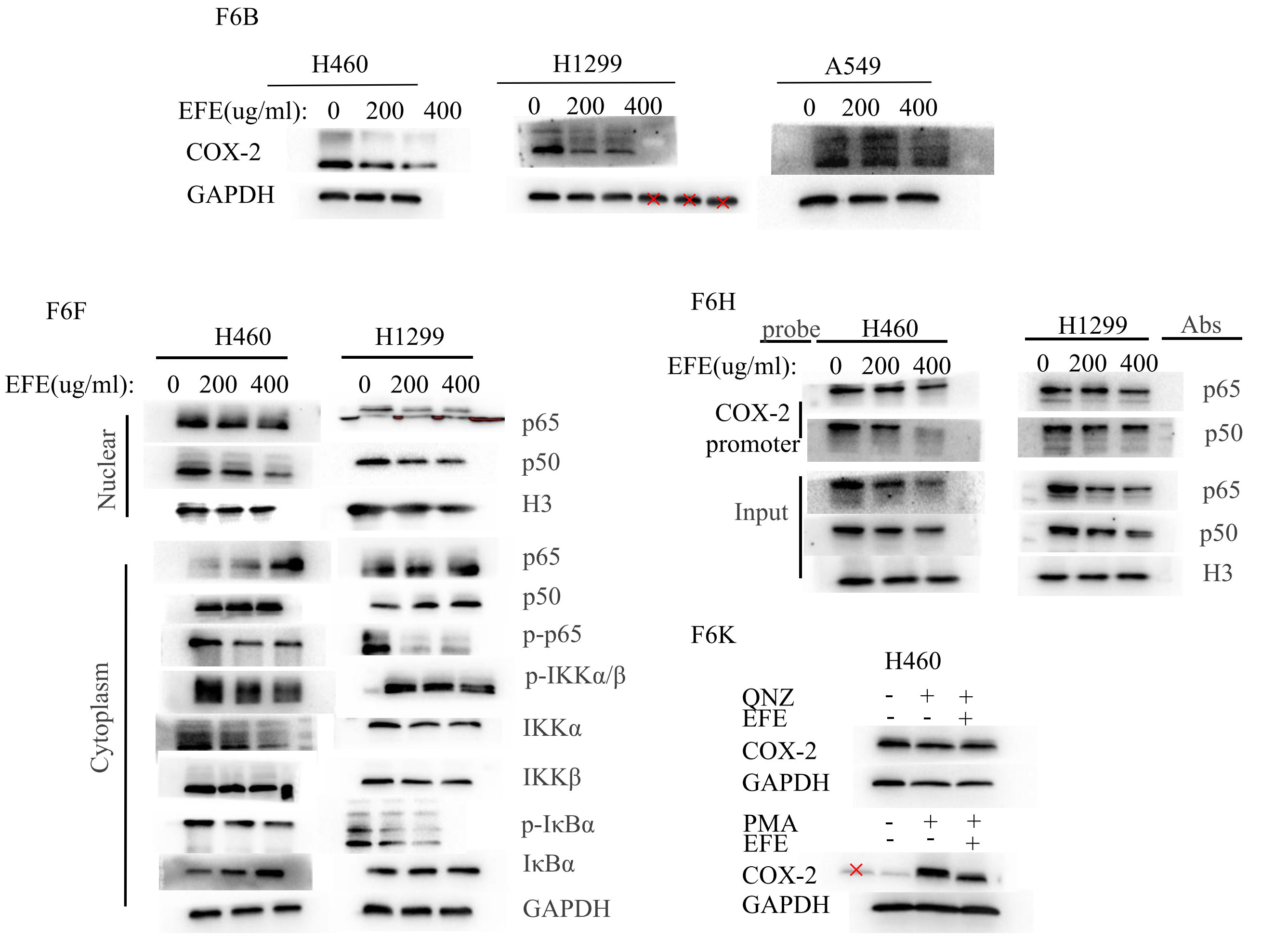


**Figure S9. Uncropped images of Western blot membranes for Figure 6.** Note that all membranes in the nuclear were re-probed for H3 and all membranes in the cytoplasm were re-probed for GAPDH, but only one H3/GAPDH representative is shown in Figure 6. All proteins were quantified using their respective H3/GAPDH for the corresponding membranes. Signal quantifications are provided in Figure 6.

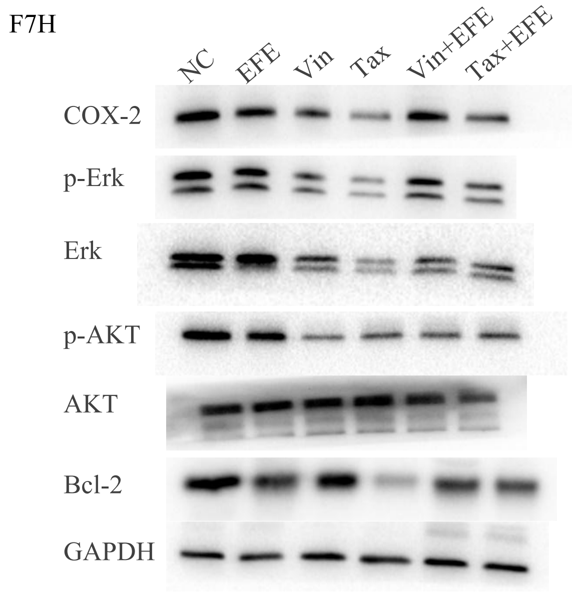


**Figure S10. Uncropped images of Western blot membranes for Figure 7.** Note that all membranes were re-probed for GAPDH, but only one GAPDH representative is shown in Figure 7. All proteins were quantified using their respective GAPDH for the corresponding membranes. Signal quantifications are provided in Figure 7.

SF1B

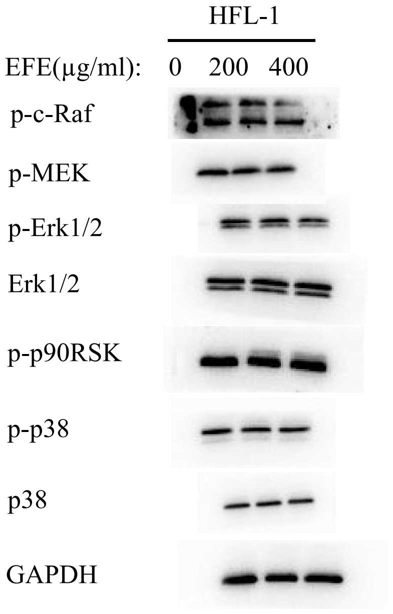


SF1C

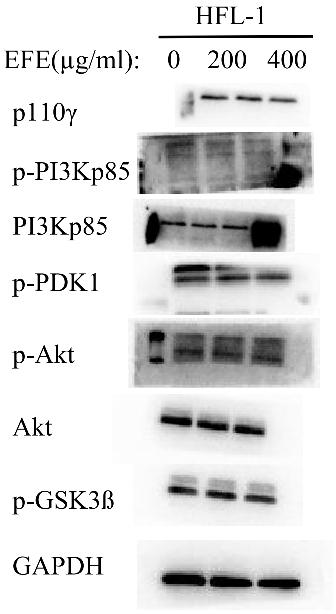


**Figure S11. Uncropped images of Western blot membranes for Figure S1.** Note that all membranes were re-probed for GAPDH, but only one GAPDH representative is shown in Supplementary Figure 1. All proteins were quantified using their respective GAPDH for the corresponding membranes. Signal quantifications are provided in Figure S1.
