# Supplementary material for: Lumbrokinase Extracted from Earthworms Synergizes with Bevacizumab and Chemotherapeutics in Treating Non-Small Cell Lung Cancer by Targeted Inactivation of BPTF/VEGF and NF-κB/COX-2 Signaling
Source: Biomolecules. 2024 Jun 23;14(7):741. doi: 10.3390/biom14070741 (PMC11274885; doi:10.3390/biom14070741)
Supplement: Supplementary file 1 [file biomolecules-14-00741-s001.zip › biomolecules-3011215-supplementary/biomolecules-3011215-supplementary.pdf]

Supplementary Figures

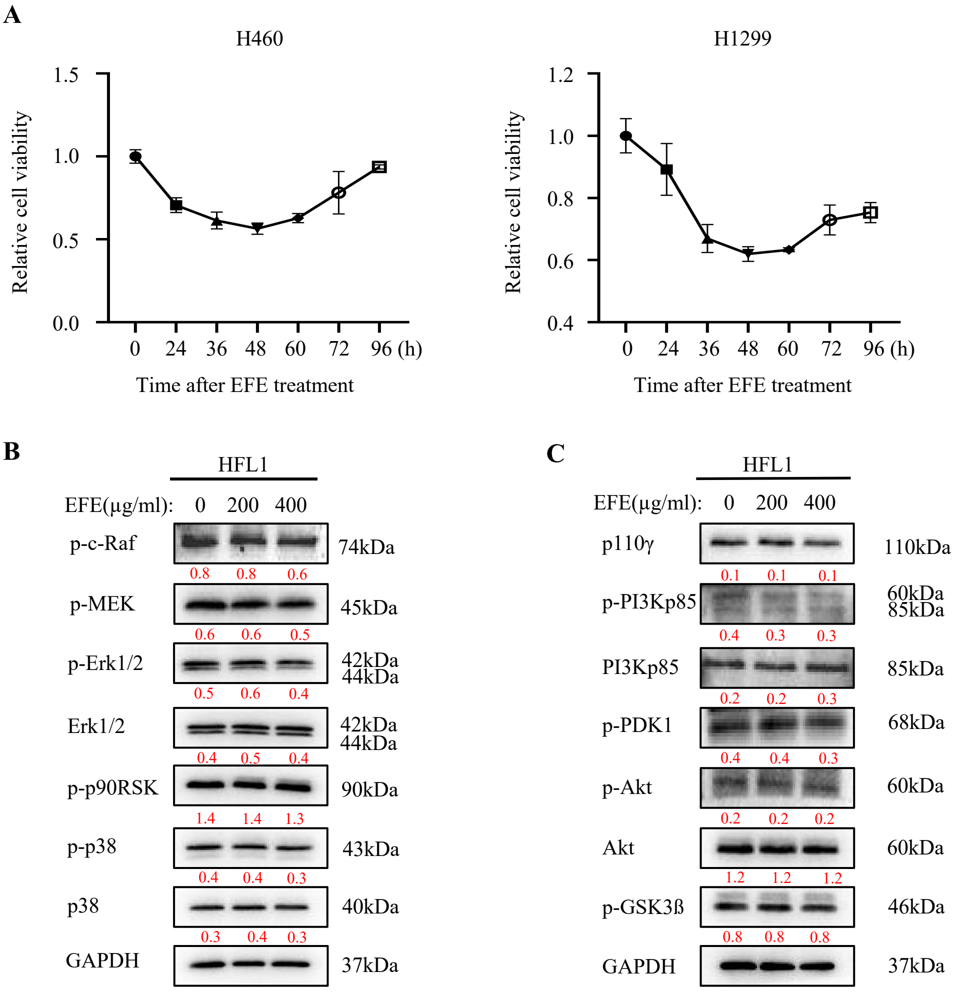

Figure S1. Lumbrokinase was cytotoxic to NSCLC cells but not to normal lung cell.

(A) H460 and H1299 cells were treated respectively with 400 μg/ml EFE for different time, and the cell viability was tested by MTT assay. (B) HFL1 cells were treated with two doses of EFE for 48 h, and the expressions of the key proteins in MAPK/Erk signaling pathway were detected by western blot. (C) The expressions of the key proteins in PI3K/Akt signaling pathway were detected in HFL1 cells with EFE treatment by western blot.

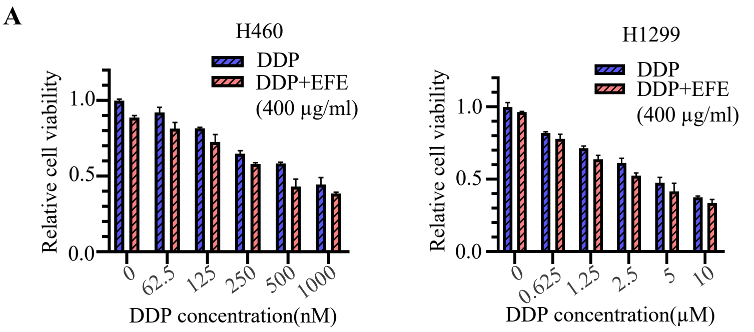

B

Cells	IC50 DDP	IC50 DDP+EFE (400 μg/ml）	Folds
H460	719.90 nM	439.93 nM	1.64
H1299	4.66 μM	3.22 μM	1.45

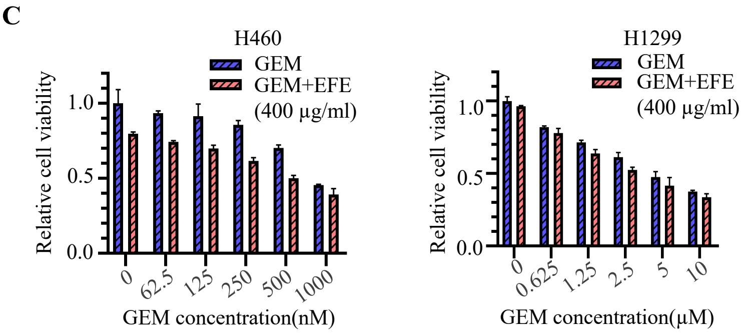

D

Cells	IC50 GEM	IC50 GEM+EFE (400 μg/ml）	Folds
H460	580.42 nM	276.82 nM	2.10
H1299	9.30 μM	5.43 μM	1.71

Figure S2. Lumbrokinase sensitized NSCLC cells to cisplatin and gemcitabine treatment.

(A) H460 and H1299 cells were treated respectively with indicated doses of cisplatin alone or cisplatin combined with 400 μg/ml EFE for 48 h, and the cell viability was tested by MTT assay. (B) IC50 values of cisplatin were calculated by CVXPT32 upon co-administration with EFE or not. (C) H460 and H1299 cells were treated respectively with indicated doses of gemcitabine alone or gemcitabine combined with 400 μg/ml EFE for 48 h, and the cell viability was tested by MTT assay. (D) IC50 values of gemcitabine were calculated by CVXPT32 upon co-administration with EFE or not.

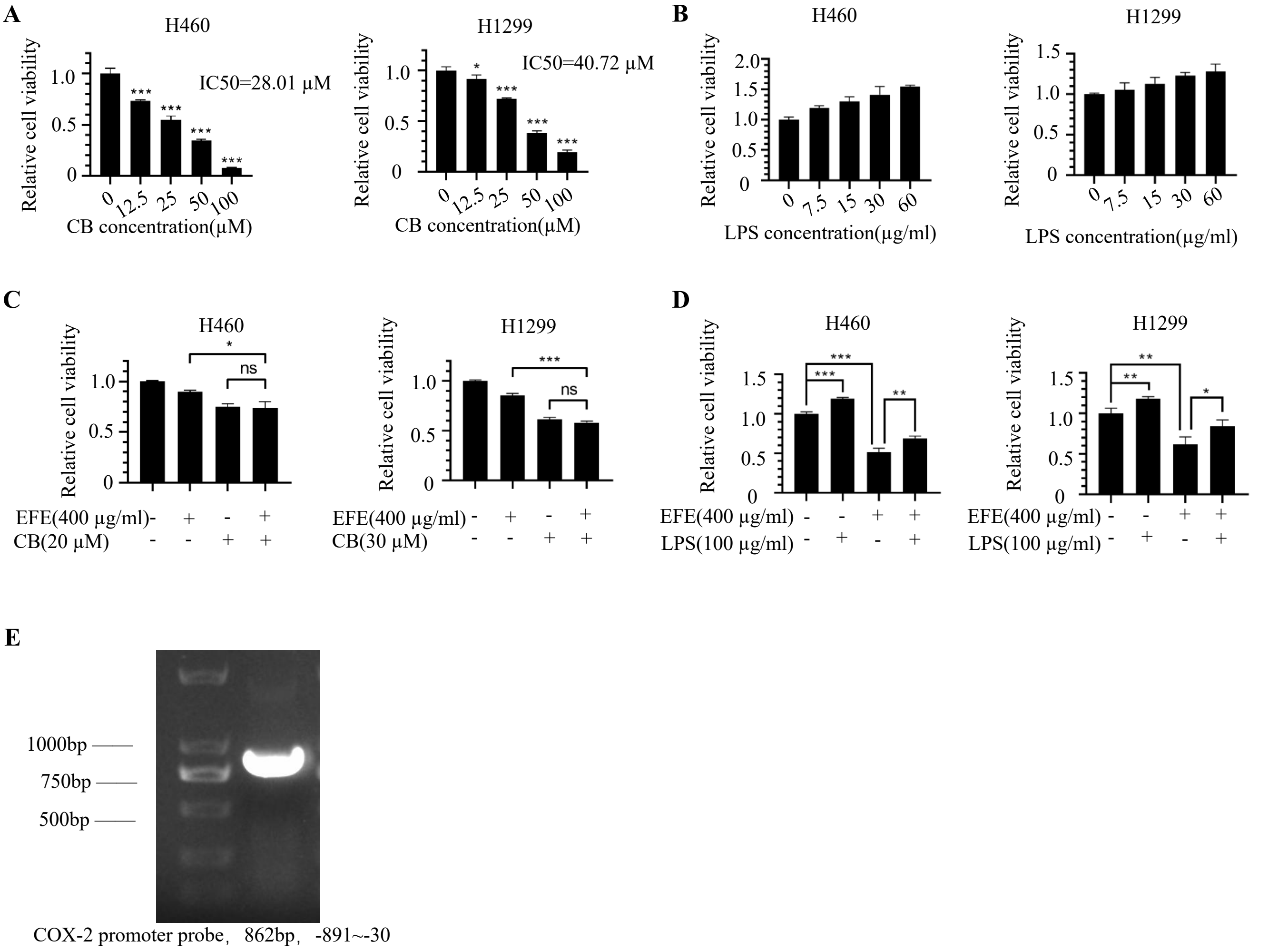

Figure S3. Lumbrokinase sensitized NSCLC cells to chemotherapeutics by targeting NF-κB/COX-2 signaling. (A) The cell viability of H460 and H1299 cells under different doses of CB treatment was tested by MTT assay and the value of IC50 was calculated by CVXPT32. (B) The cell viability of H460 and H1299 cells under different doses of LPS treatment was tested by MTT assay. (C) H460 and H1299 cells were treated with EFE (400 μg/ml) or CB (20 μM) alone, or their combination for 48 h, and then the cell viability was tested by MTT assay. (D) H460 and H1299 cells were treated with EFE (400 μg/ml) or LPS (100 μg/ml) alone, or their combination for 48 h, and then the cell viability was tested by MTT assay. (E) A biotinylated DNA probe of COX2 promoter for DNA pull down assay was synthesized by RT-PCR. The level of significance was indicated by * $p < 0.05$, ** $p < 0.01$, and *** $p < 0.001$, and ns means no statistical significance.
